# The influence of coiled-coil motif of serine recombinase toward the directionality regulation

**DOI:** 10.1016/j.bpj.2023.11.009

**Published:** 2023-11-16

**Authors:** Yei-Wei Chen, Bo-Yu Su, Gregory D. Van Duyne, Paul Fogg, Hsiu-Fang Fan

**Affiliations:** 1 Institute of Medical Science and Technology, National Sun Yat-sen University, Kaohsiung, Taiwan; 2 Department of Chemistry, National Sun Yat-sen University, Kaohsiung, Taiwan; 3 Aerosol Science Research Center, National Sun Yat-sen University, Kaohsiung, Taiwan; 4 Department of Life Sciences and Institute of Genome Sciences, National Yang-Ming University, Taipei, Taiwan; 5 Perelman School of Medicine, University of Pennsylvania, Philadelphia, Pennsylvania; 6 Biology Department and York Biomedical Research Institute (YBRI), University of York, York, United Kingdom

## Abstract

Serine integrases promote the recombination of two complementary DNA sequences, *attP* and *attB*, to create hybrid sequences, *attL* and *attR*. The reaction is unidirectional in the absence of an accessory protein called recombination directionality factor. We utilized tethered particle motion (TPM) experiments to investigate the reaction behaviors of two model serine integrases from *Listeria innocua* phage *LI* and *Streptomyces coelicolor* phage C31. Detailed kinetic analyses of wild-type and mutant proteins were carried out to verify the mechanisms of recombination directionality. In particular, we assessed the influence of a coiled-coil motif (CC) that is conserved in the C-terminal domain of serine integrases and is an important prerequisite for efficient recombination. Compared to wild type, we found that CC deletions in both serine integrases reduced the overall abundance of integrase (Int) *att-*site complexes and favored the formation of nonproductive complexes over recombination-competent complexes. Furthermore, the rate at which CC mutants formed productive synaptic complexes and disassembled aberrant nonproductive complexes was significantly reduced. It is notable that while the φC31 Int CC is essential for recombination, the *LI* Int CC plays an auxiliary role for recombination to stabilize protein-protein interactions and to control the directionality of the reaction.

## Graphical abstract

**Figure:**
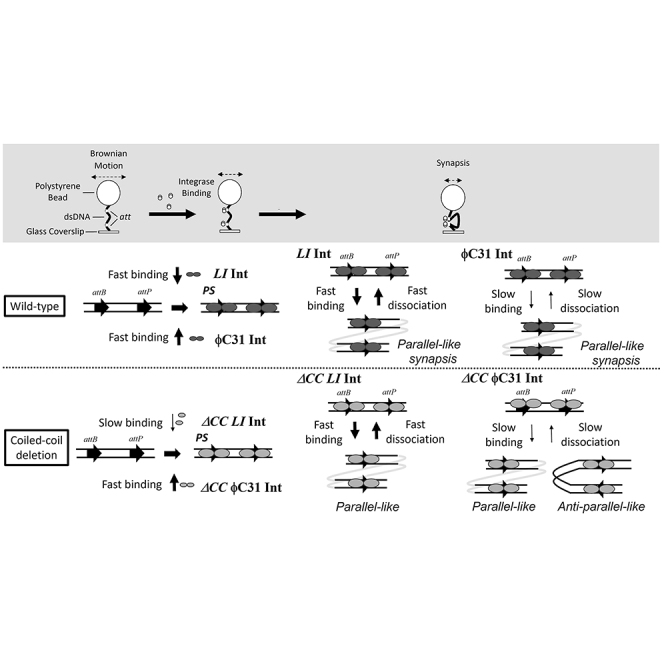

## Significance

In this project, two model serine integrases from *Listeria innocua* phage *LI* and *Streptomyces coelicolor* phage C31 were investigated. In terms of kinetics, deleting the coiled-coil (CC) motifs in both serine integrases leads to a slower association of synaptic complexes and a slower dissociation of nonproductive complexes (NP → S). Regarding thermodynamics, the deletion of CC motifs modulates the relative abundance of Int-bound att-site complexes (both *att*B × *att*P and *att*R × *att*L), favoring the formation of NP complexes over recombination-competent presynaptic complexes in both serine integrases. The CC motifs in *LI* integrase play an auxiliary role in strengthening synaptic interface interactions, and their absence allows *LI* integrase to exhibit similar recombination activity on both *att*B × *att*P and *att*L × *att*R systems. In contrast, the CC motifs of φC31 integrase are essential prerequisites for synaptic interface formation, and the lack of a CC domain not only severely inhibits synapsis but also disrupts the proper orientation of att sites during synapsis, resulting in impaired recombination efficiency for φC31 integrase.

## Introduction

Site-specific recombinases (SSRs) are enzymes that recognize and rearrange specific DNA substrates (*att* sites), a characteristic that has long been exploited for synthetic biology and genome engineering applications (1,2). SSRs can be categorized into two major families, Tyrosine and Serine families, depending on the identity of the catalytic nucleophile (1,2). Both families catalyze the cleavage and exchange of DNA strands but do not share significant sequence similarity or mechanisms of action (3). Serine recombinases generally have simpler DNA *att* site and co-factor requirements and are thus more widely used in biotechnology (4).

**Figure 1:**
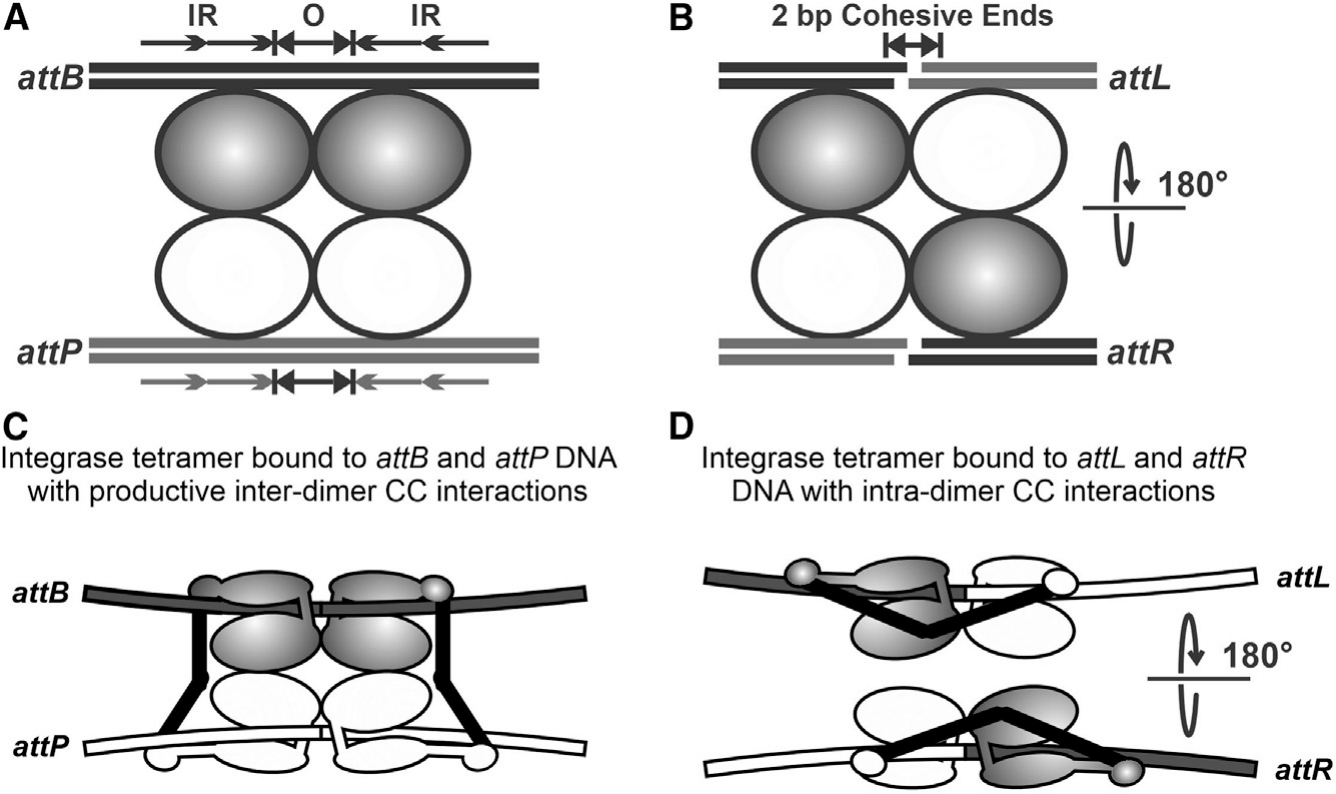
Schematic of the serine integrase site-specific recombination process. (*A*) An integrase tetramer, with the two constituent dimers bound to the *attB* or *attP* sites. The simple attachment sites consist of a short complementary overlap sequence (O) flanked by inverted repeats (IR). (*B*) The integrases cleave both strands of the *attB* and *attP* sites at the overlap region, producing 2-bp cohesive ends. The integrases undergo 180° subunit rotation, which leads to exchange of the *att*B/P half sites. The DNA is ligated to form two new recombinant attachment sites, *att*L and *att*R. (*C*) When integrase is bound to *att*B or *att*P, the C-terminal CC motifs (shown in *black*) project away from the DNA and facilitate interdimer interactions that promote recombination. (*D*) When integrase is bound to *att*L or *att*R, the CC motif takes up a conformation that is favorable for intradimer interactions that inhibit recombination. The recombination directionality factor is thought to bind at the base of the coiled coil and to alter its trajectory to a recombination-competent conformation.

All serine recombinases possess a conserved N-terminal catalytic domain, responsible for DNA cleavage and ligation (5,6), but the size of the C-terminal domain is variable. Resolvase/invertases have a small (∼60 amino acids (aa)) C-terminal domain and can perform DNA recombination in a bidirectional manner (7). The large serine recombinases (LSRs) are a diverse family with an extended (∼300–500 aa in length) C-terminal domain (5). The LSR family includes well-characterized phage integrases from *Streptomyces* phage φC31, mycobacteriophage Bxb1, *Listeria innocua* prophage (*LI* Int), and the closely related *Listeria* phage A118 (8,9,10,11). The current model for serine integrase-mediated recombination is that integrase dimers bind to a specific sequence in the phage (*attP*) and the host (*attB*) genome to initiate the integration reaction (Fig. 1). Both *att* sites are ∼40–50 bp in length and are composed of inverted repeats flanking a short overlap region that is identical in *attB* and *attP* (5,12). Synapsis occurs between Int_2_-*attP* and Int_2_*-attB* to form a tetrameric intermediate. The serine integrase then simultaneously cleaves all four DNA strands in the center of the *att* recognition sites, yielding cohesive ends with 2-bp overhangs. The half sites are rotated 180° relative to one another and then ligated to form recombinant *att* sites—*attL* and *attR* (13,14). In the presence of integrase alone the recombination reaction is entirely unidirectional, i.e., *attB* × *attP* ⇒ *attL* × *attR*, although there are some minor exceptions (15,16).

Data for *LI* integrase showed that its structural conformation is dependent upon the *att* site to which it is bound and that conformational changes in the integrase subunits affect the stability of synaptic complexes and, thus, recombination efficiency (17,18). When integrase binds to *attB* or *attP*, a C-terminal coiled-coil (CC) domain extends away from the main protein-DNA complex. The CC facilitates interdimer interactions between the integrases bound to *attB* and *attP* (Fig. 1), which in turn favors synapsis and successful recombination. On the other hand, when integrase binds to *attL* or *attR*, the CC domains promote the formation of stable intradimer interactions, which inhibit synapsis and thus block recombination (Fig. 1). Interruption of *LI* Int dimerization by deletion of the CC domain or mutation of key residues in the CC dimer interface impairs recombination directionality control (18). Meanwhile, multiple mutants of φC31 integrase capable of promiscuous *attB* × *attP* and *attL* × *attR* recombination are all located on one face of the coiled-coil motif, reinforcing the conserved importance of the CC in the regulation of LSR directionality (19). However, a conspicuous difference between φC31 and *LI* integrase is that φC31 Int ΔCC is completely inactive, whereas *LI* Int ΔCC retains recombination activity (18,20).

For wild-type (wt) serine integrases to efficiently catalyze *attL* × *attR* recombination, an additional phage-encoded recombination directionality factor (RDF) is required (21). The RDF protein alters recombination directionality exclusively via direct interaction with the integrase, and no DNA binding is required (10,20). It has been suggested that when integrase is bound to *attL* and *attR*, the RDF protein promotes the reconfiguration of CC motifs in a way that facilitates interdimer interactions and subsequent synapsis (10,20). So far, the available data obtained from DNA binding, recombination efficiency and mutations of *att* sites, integrases, and RDF proteins is consistent with this architectural model (17,19,20,22,23,24). Indeed, data for φC31 demonstrate that the RDF binds to a putative hinge region at the base of the integrase CC motif, and several amino acids in this region are important for recombination efficiency (20,24).

Most data for the mechanism of serine integrase recombination are derived from end-point assessments of recombination efficiency and accumulation of reaction intermediates. We have previously used single-molecule approaches to calculate the kinetics of individual steps of the recombination reaction and to assess the formation and resolution of reaction intermediates in real time (25,26,27,28,29,30). Recently, we have focused on the RDF-dependent and RDF-independent regulation mechanisms of serine recombinases using φC31 integrase (26,28). It is known that the hyperactive φC31 integrase E449K can catalyze RDF-independent *attL* × *attR* recombination without loss of *attB* × *attP* recombination capabilities. Our data supported the hypothesis that the E449K mutation promotes the formation of presynaptic (PS) complexes over nonproductively bound complexes (a dead-end intermediate). Moreover, E449K accelerates the dissociation of nonproductively bound complexes and inactive synaptic complexes, both of which accelerate the rate of successful recombination (28).

Here, the single-molecule tethered particle method was adapted to investigate the influence of CC domain interactions of *Listeria innocua* integrase and φC31 integrase on the directionality regulation mechanism. We sought to investigate the influence of CC dimer interface mutations in individual recombination steps, to distinguish the topological preference of active synapses from inactive ones, and to address the difference of CC motif regulatory roles between *LI* Int and φC31 Int systems.

## Materials and methods

### Proteins

*LI* integrase expression plasmids used in this study are the same as in (18). The *LI* integrases and mutant proteins were expressed in *Escherichia coli* (BL21, DE3) and purified according to published procedures (10,18). φC31 integrase and ΔCC *φC31* protein plasmids used in this study are the same as in (20). The φC31 integrase and ΔCC *φC31* proteins were expressed in *Escherichia coli* (BL21, DE3) and purified according to published procedures (21,31).

### DNA substrates

The *att-*site-containing plasmids were constructed in the PL451 vector (obtained from American Type Culture Collection, Manassas, Virginia). The *attB* or the *attL* site was cloned between the SalI and BspEI sites of the vector. The *attP* or *attR* site was inserted between the BssHII and BamHI sites. The sequences of the *att* sites are listed in Table S1.

The 1303-bp-long recombination substrates and control molecules with a single *att* site were prepared by PCR amplification of PL451-derived plasmids containing *attB*-*attP*, *attL*-*attR*, *attB*, or *attP* (Table S2). A 551-bp mimic of the linear excision product of *attB* × *attP* recombination was obtained from the plasmid containing head-to-tail *attL*-*attR*. For preparing the 1303-bp control DNA without an *att* site, the template was pBR322 DNA. The primer pair in each PCR reaction contained a 5′-digoxigenin label in one and a 5′-biotin label in the other (Table S2). All PCR reactions were conducted with Pfu polymerase according to the vendor’s instructions (MDBio, Taipei, Taiwan).

### Single-molecule TPM measurement and data analysis

One end of the DNA molecules was anchored on a glass coverslip by digoxigenin-anti-digoxigenin interactions. These molecules were tethered at the other end to polystyrene beads (200 nm in diameter) by biotin-streptavidin interactions. The details of the TPM analysis, including reaction chambers, sample preparation, and the criteria for eliminating aberrantly behaved molecules from data analysis, have been previously described (27,32). All data presented here were smoothed using a five-point adjacent averaging algorithm. The Brownian motion (BM) histograms were fitted using either a single-peak or bimodal distribution, where the peak distribution was described by a normal distribution equation. We employed single-peak and double-peak fitting algorithms to model the BM distribution and compared the results using built-in tests in Origin 2023, which included the F-test, Akaike information criterion, and Bayesian information criterion, to determine the preferred fitting model. The BM amplitude values are expressed as the mean ± 1 SD.

The dwell-time plots (Figs. 4 *C* and *D*, 5 *C* and *D*, and 6 *B* and *D*) in the figures displaying the TPM data were created by pooling the cumulative data from five to seven repetitions of the experiment. To calculate the dwell-time estimates of PS complex and nonproductive (NP) complex formation, only the initial events were counted. For all other dwell times, the analysis pooled multiple transition events undergone by a single molecule. The *N* values in each dwell-time histogram (Figs. 4, 5, and 6) denote the total number of transition events from the recorded time traces.

We generated histograms using a cumulative counting approach to prevent the influence of binning sizes. Previous studies have shown a remarkable similarity between the values derived from cumulative counting histograms and the fitting values obtained through an unbinned maximum-likelihood estimation approach (27). The dwell-time histograms were fitted to a single-exponential algorithm (*y* = *A*_1_ × e^(−*k*1×*t*)^) by Origin 8.0 software. The goodness of fit was 0.87 ≤ *R*^2^ ≤ 1.0.

### Recombination reactions investigated by TPM experiments

Based on previous studies, it has been pointed out that removal of the CC motif resulted in a weaker monomer-dimer dissociation constant *K*_D_ = 5.7 μM for ΔCC *LI* Int in comparison to *K*_D_ = 0.032 μM for full-length wt *LI* Int (18), even though a higher affinity to *att*P, with *K*_D_ = 6.9 ± 0.7 nM, is reported for full-length wt *LI* Int (12). The binding of one LI Int dimer to *att* site cannot be differentiated due to the spatial limitation of the TPM assay (Fig. S1), and a similar observation has been reported for φC31 integrase (26). For wt *LI* integrase proteins, recombination activity is only detectable at concentrations higher than 50 nM, whereas for *LI* Int CC interaction mutants, recombination activity is only observed at a concentration higher than 200 nM. Here, the concentrations of *LI* integrase proteins (wt and S10A) were set at 100 nM and 200 nM for the *attB* × *attP* and *attL* × *attR* systems, respectively, in order to acquire more response events. For the *LI* Int CC mutants, the concentration was set at 200 or 400 nM in order to acquire more response events. For the *LI* integrase system, the reaction chamber (22°C) containing the tethered DNA molecules was buffered with 20 mM Tris-HCl (pH 8.0), 150 mM KCl, 5% glycerol, 5 mM MgCl_2_, 5 mM dithiothreitol, and 2 mg/mL bovine serum albumin (BSA). *LI* integrase (200 nM) was added in the same buffer to initiate the reaction. At the end of the 30-min incubation period, 100 μL of 0.05% sodium dodecyl sulfate (SDS) in the reaction buffer was poured into the chamber to quench the reaction.

As for φC31 integrase proteins, the choice of 20 nM concentration of integrase, which is close the 5–20 nM *K*_D_ reported in the absence of ethylenediaminetetraacetic acid (33), was based on the optimization for TPM observations without nonspecific DNA association and without causing molecules to stick to the surface (26,28). For the φC31 integrase system, the reaction chamber (22°C) containing the tethered DNA molecules was buffered with 10 mM Tris-HCl (pH 8.0), 100 mM NaCl, 4.5% glycerol, 5 mM dithiothreitol, and 2 mg/mL BSA. φC31 integrase (20 nM) was added in the same buffer to initiate the reaction. At the end of the 30-min incubation period, 100 μL of 0.05% SDS in the reaction buffer was poured into the chamber to quench the reaction.

## Results

### Rationale of the single-molecule TPM analysis of recombination by serine integrases

We previously used the single-molecule TPM analysis technique to investigate the φC31 integrase-mediated site-specific recombination process from beginning to end in real time, and the influence of the E449K hyperactive mutation on the directionality of recombination (26,28). However, the lack of complete structural information for φC31 integrase limited our ability to fully understand the directionality regulation mechanism. Structural data are available for the analogous *LI* Int proteins bound to *att*-site DNA, and these data support a regulatory model whereby CC interactions between integrase subunits mediate recombination directionality (10,12,18). Here, the same TPM experimental designs and analysis procedures used for φC31 integrase were applied to *LI* integrase and its mutants to investigate the reaction mechanism and control of directionality.

In the TPM system, we can monitor the binding and recombination of substrate DNA by serine recombinase proteins over time. One end of the DNA is anchored to a glass coverslip by digoxigenin-anti-digoxigenin interactions, and the other end is bound to a 200-nm diameter polystyrene bead by biotin-streptavidin interactions (25,26,27,28,30). The BM amplitude of the polystyrene bead is proportional to the length of the DNA and/or the action of proteins bound to it. By analyzing the changes in BM amplitude, we can determine the formation of different protein-DNA complexes or successful recombination of the DNA.

**Figure 2:**
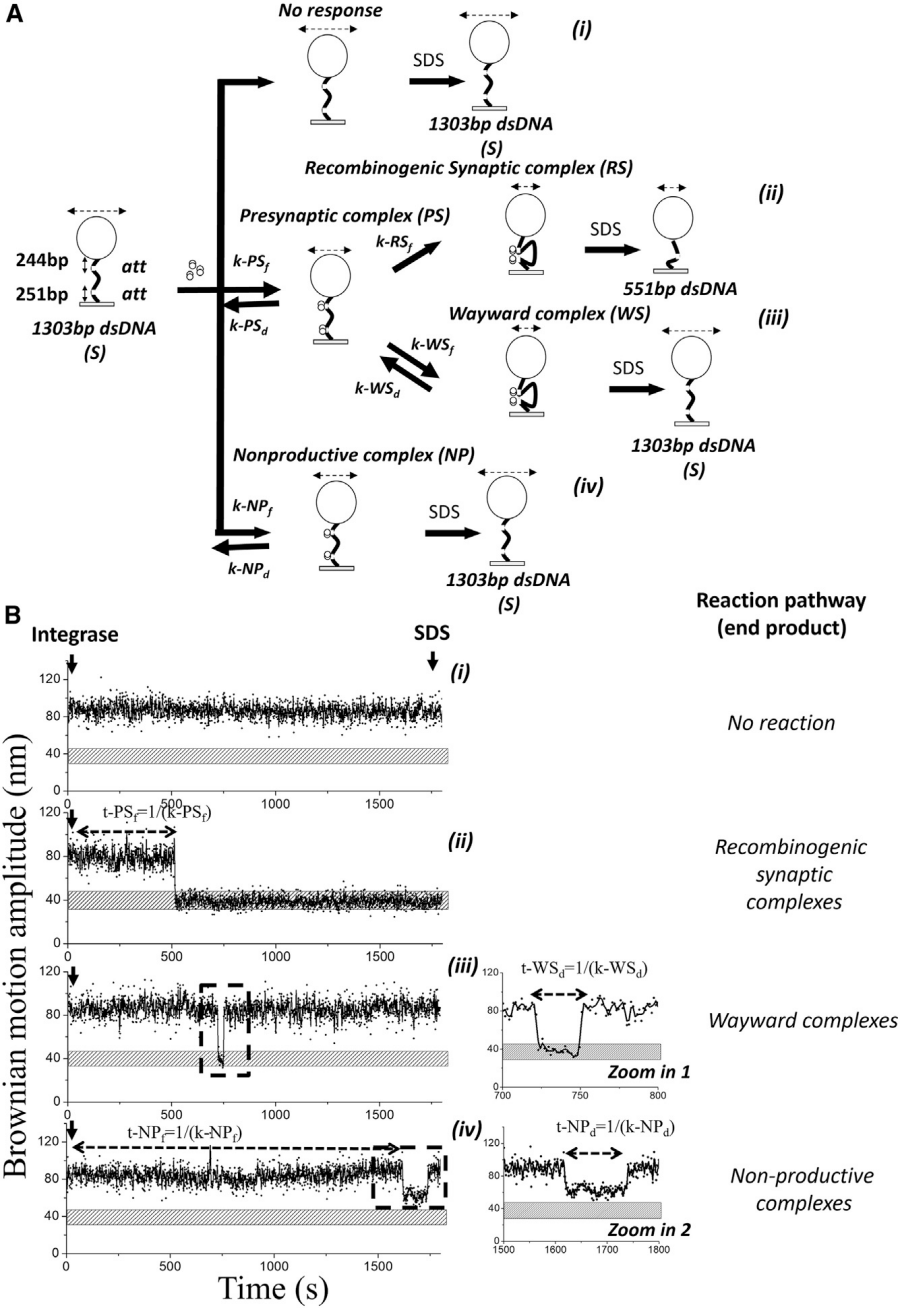
Tethered particle motion (TPM) assay to investigate *LI* Int-mediated site-specific recombination process. (*A*) Diagram of the DNA recombination pathways in the TPM experimental setup. One end of a 1303-bp dsDNA substrate is anchored to a glass slide, and the other end is tethered to a 220-nm polystyrene bead (shown as a *sphere*). The Brownian motion amplitude (BM amplitude) of the bead is represented above the sphere by double-headed arrows. Binding of integrase dimers (*paired circles*) to the DNA *att* sites or recombination of the DNA decreases the BM signal, and the extent of this change in BM amplitude corresponds to the annotated reaction intermediates or the reaction product. The individual complexes are described in the text. The rates of complex formation or dissociation are labeled on each reaction arrow, and the corresponding values are summarized in Table 1. The experiment is terminated by addition of SDS detergent. (*B*) Typical time traces illustrating the behavior described in (*Ai*)*–*(*Aiv*) are shown. The horizontal stippled bars in indicate the BM amplitude of expected excision products, 551 bp dsDNA. The dwell times used to calculate rate constants are indicated by double-headed dashed arrows, with the corresponding formulas labeled above the line. The enlarged “zoom” insets show the regions used to calculate the dissociation rate constants. More representative time traces can be found in Figs. S11 and S12.

For 1303-bp double-stranded DNA (dsDNA) molecules with *att* sites oriented in a head-to-tail orientation, up to four distinct reaction states can be classified (Figs. 2 *A* and S1): (i) “No response,” i.e., there is no significant change from the starting BM amplitude (88.8 ± 4.5 nm) after addition of integrase. (ii) “Recombinogenic synaptic (RS) complexes,” i.e., the integrase initially binds to both *att* sites, forming PS complexes, and successfully forms an RS complex with a low BM amplitude of 41.4 ± 3.2 nm. This low amplitude persists even after the SDS challenge, indicating the successful recombination of the 551-bp DNA. (iii) “Wayward synaptic (WS) complexes,” i.e., the integrase initially binds on both *att* sites to form PS complexes and attempts to form a synaptic complex, but the reaction remains incomplete, indicated by a transient change in BM amplitudes that briefly (∼10–20 s) extend down to 41 ± 9 nm, corresponding to the amplitudes of looped synaptic complexes. However, the nonsuccessful complexes (WS) exhibit a high BM amplitude of 88.8 ± 4.5 nm, corresponding to the unmodified 1303-bp dsDNA substrate after the SDS challenge. (iv) “Nonproductive (NP) complexes,” i.e., the NP complexes display an average BM amplitude of 62.7 ± 5.6 nm. These complexes represent DNA fragments where Int is bound to both *att* sites but does not form looped synaptic complexes or generate recombinant products. After SDS challenge, the BM amplitude returns to the value corresponding to unmodified substrate DNA (88.8 ± 4.5 nm). Four representative BM time traces corresponding to the above conditions are listed in Fig. 2 *Bi–iv*. The rate constants of complex formation or decomposition were calculated by pooling the dwell times for each reaction state and fitting the data with a single-exponential decay model (30).

### The recombination behaviors of wt *LI* integrase and mutant *LI* integrase

**Figure 3:**
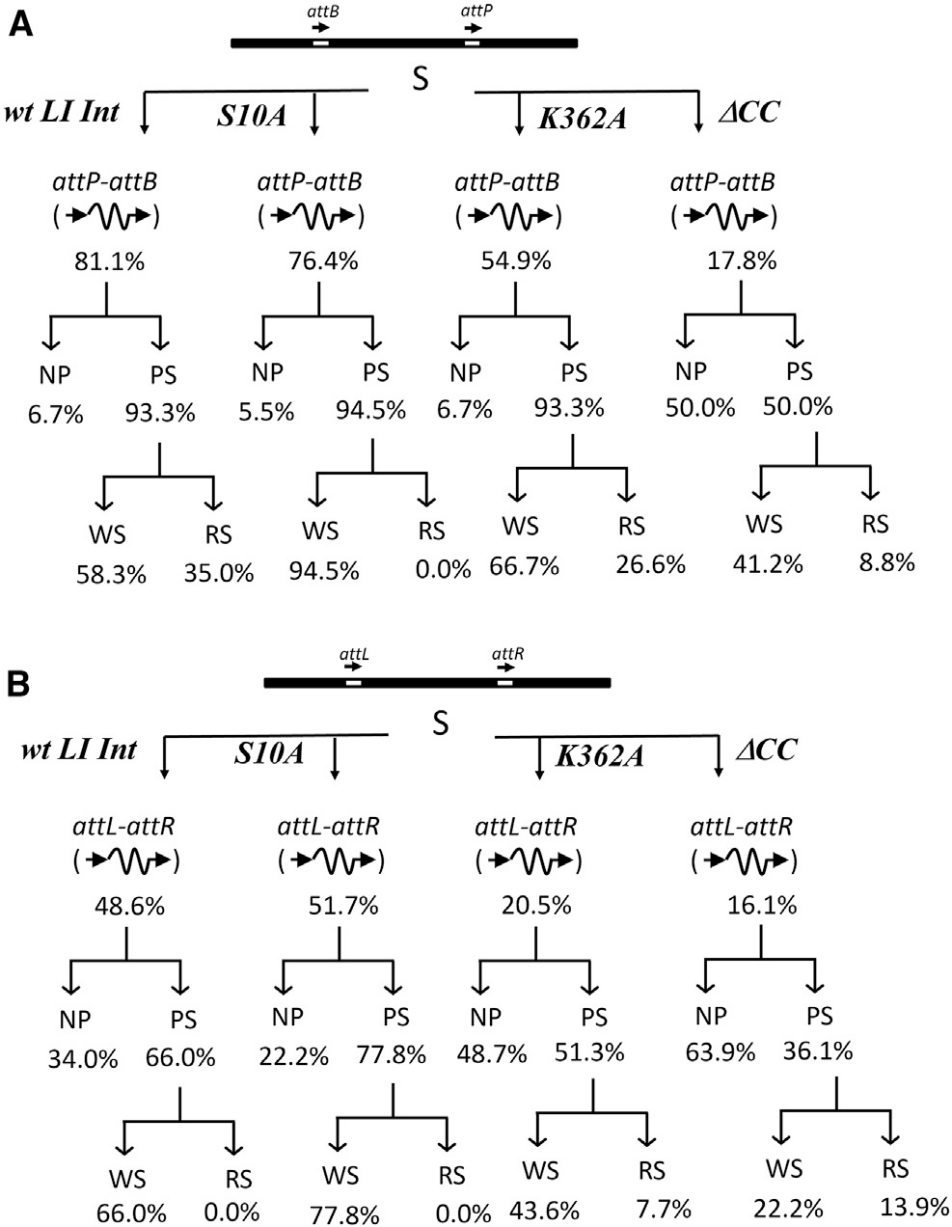
Characterization of *attB × attP* and *attL × attR* recombination by *LI* integrase. Abundance of different reaction behaviors of wt *LI* integrase, S10A *LI* integrase, K362A *LI* integrase, or ΔCC *LI* integrase with DNA containing (*A*) *attB* × *attP* sites in head-to-tail orientation or (*B*) *attL* × *attR* sites in head-to-tail orientation. The values stated are expressed as percentages of the absolute values observed in the preceding tier. Simplified diagrams of *att*-site composition and orientation are shown. “S” represents substrate DNA.

Previous studies have proposed that the integrase C-terminal CC motif plays an important role in the regulation of recombination by stabilizing Int dimers formed by N-terminal domain interactions (18,19,24,34). Moreover, the CC dimerization promotes integration and inhibits excision during the serine integrase recombination process (18). Here, we investigated the behavior of wt *LI* integrase and derivative mutants with single-molecule TPM experiments (Fig. S2). Comparable DNA-binding activities were observed for wt *LI* integrase and *LI* Int^S10A^ of 81.1% and 76.4%, respectively, with *attB*/*attP* in head-to-tail orientation (Fig. 3 *A*). As expected, no recombination products were observed for *LI* Int^S10A^ because S10 is the catalytic nucleophile and substitution with alanine abolishes its DNA cleavage ability (18). To assess the impact of the *LI* Int CC motif on recombination directionality, we used *LI* Int ΔCC and *LI* Int^K362A^ (18,34). Int^K362^ is located within the CC and has been proposed to form salt bridges with other residues from both the same CC subunit and the partner helices, suggesting an important role in the CC dimer interface (18). In our experiments, Int^K362A^ DNA-binding activity was reduced to 54.9% accompanied with a final recombination efficiency of 14.6% (calculated as 54.9% *att*-site binding × 93.3% PS complex formation × 28.5% successfully recombined product) (Fig. 3 *A*). Meanwhile, *LI* Int ΔCC was even more severely affected, with a bound DNA fraction of 17.8% and a final recombination efficiency of only 1.6% (Fig. 3 *A*). These data are consistent with previous findings that CC mutants defective at integrase dimerization exhibit reduced integration activities (18). The above observations indicate that the reduced recombination efficiency of *LI* Int CC mutants is a result of defects at multiple stages of the reaction, i.e., DNA-binding efficiency, PS complex formation, and the final recombination step.

Similar experiments and analysis procedures were applied on 1303-bp dsDNA containing *attL* × *attR* in a head-to-tail orientation. Significantly reduced *att*-binding activities of 48.6% and 51.7% were obtained for wt *LI* Int and *LI* Int^S10A^, respectively, and both integrases failed to successfully catalyze recombination in the absence of RDF (Fig. 3 *B*). For *LI* Int^K362^, the bound DNA fraction was reduced to 20.5%; however, a final recombination efficiency of 2.6% was observed. Meanwhile, *LI* Int ΔCC possessed *attL* × *attR* DNA-binding (16.1%) and final recombination efficiency (2.2%) comparable to those observed for *LI* Int ΔCC with the *attB* × *attP* substrate (Fig. 3, *A* and *B*). These data support previous findings that the absence of the CC motif allows *LI* integrase to promiscuously recombine on both *attB* × *attP* and *attL* × *attR* substrates, albeit at low relative efficiency for both (18).

### The kinetic behaviors of wt and mutant LI integrase

**Figure 4:**
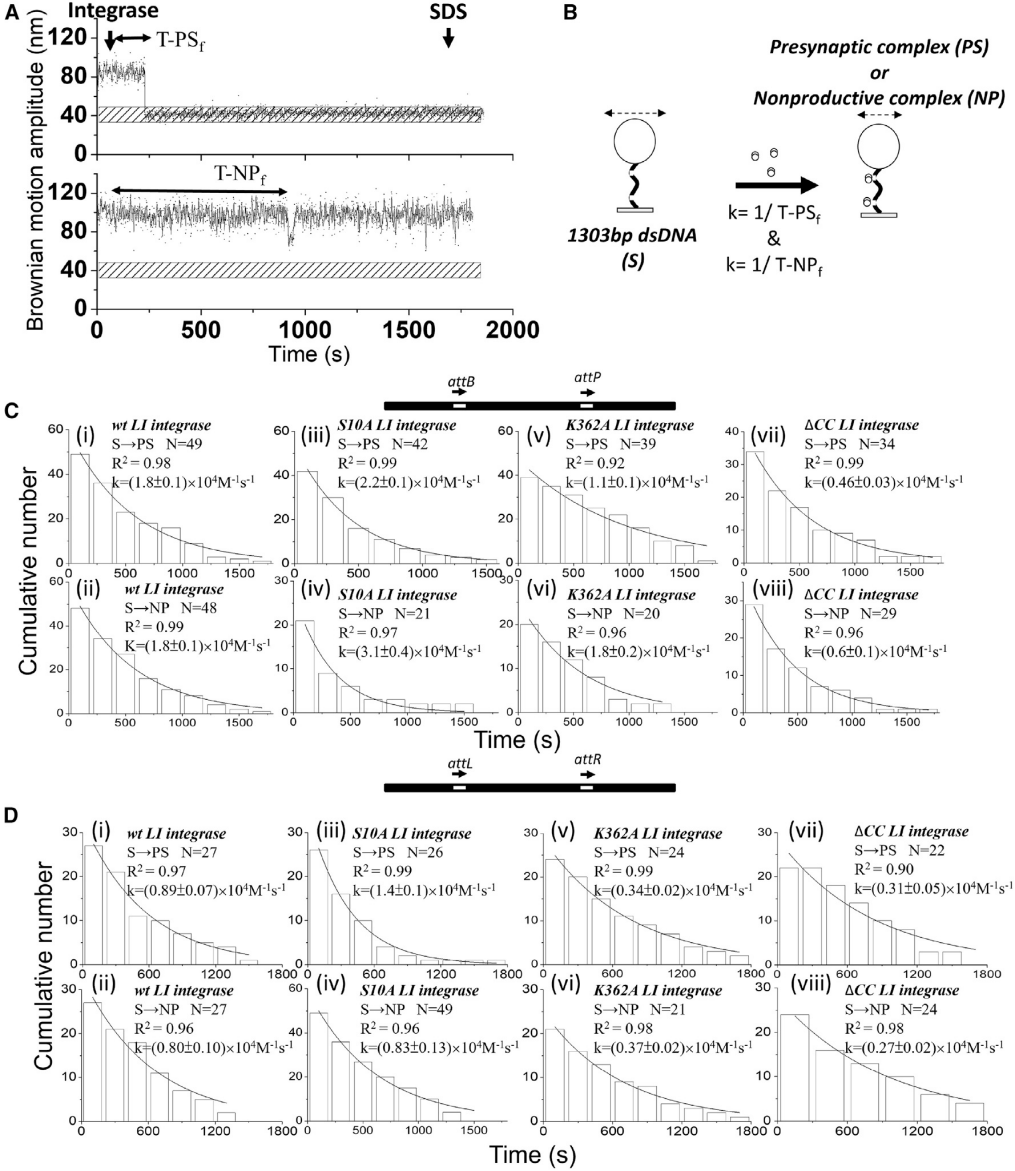
Kinetic analysis of *LI* integrase presynaptic (PS) or nonproductive (NP) complex formation. (*A*) Representative time traces for 30-min TPM reactions. The dwell time between integrase addition and the change in BM amplitude is indicated by a double-headed dashed arrow. Values for the rate of formation of PS complexes and NP complexes were pooled. Reactions were ended by addition of SDS, as annotated. The horizontal stippled bars indicate the BM amplitude of expected excision products. (*B*) Schematic illustration of PS or NP complexes. (*C* and *D*) Histograms showing the association times of PS complexes and NP complexes formed on *attB-attP* (*C*) and *attL-attR* (*D*) sites in head-to-tail orientation were obtained for wt *LI* integrase, S10A *LI* integrase, K362A *LI* integrase, and ΔCC *LI* integrase. The data were fitted to a single-exponential decay algorithm. The association rate constant was normalized to the concentration of integrase used in each condition with a unit of M^−1^ s^−1^. *N* annotated on each graph is the number of molecules observed. All data were fitted by using 8.0 Origin. The error is in 95.5% confidence interval (CI). All fitting values are listed in Table 1.

**Figure 5:**
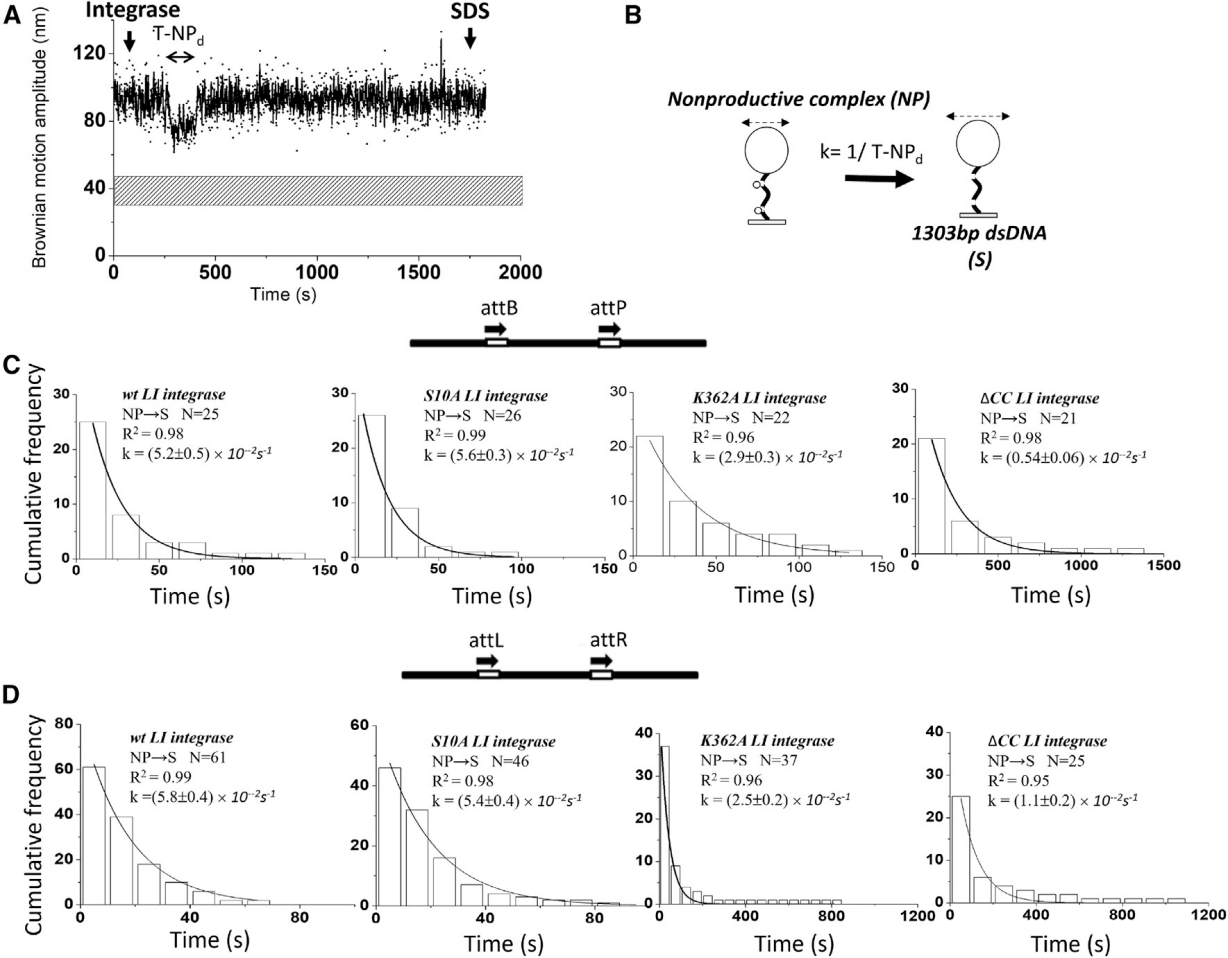
Kinetic analysis of LI integrase nonproductive (NP) complex dissociation. (*A*) Representative time traces for 30-min TPM reactions. Measurements of the dwell time in the NP state were pooled and fitted with single-exponential decay model. (*B*) Schematic illustration of an NP complex. (*C* and *D*) Histograms showing the disassociation times of NP complexes formed on *attB-attP* (*C*) and *attL-attR* (*D*) sites in head-to-tail orientation were obtained for wt *LI* integrase, S10A *LI* integrase, K362A *LI* integrase, and ΔCC *LI* integrase. The data were fitted to a single-exponential decay algorithm. *N* annotated on each graph is the number of molecules observed. All data were fitted by using 8.0 Origin. The error is in 95.5% CI.

In previous studies, it has been reported that although *LI* Int ΔCC can recombine both *attB* × *attP* and *attL* × *attR* substrates, site alignment during synapsis is biased against the formation of successful recombinant products (10,18). To assess how the CC domains of *LI* Int affect the directionality of recombination, kinetics analysis was applied to obtain rate constants for the individual steps depicted in Fig. 2 *A* (analysis data are shown in Figs. 4, 5, and S3–S6; Table 1;). With the TPM assay, it is difficult to differentiate the binding of neither one *LI* Int monomer nor one *LI* Int dimer to a single *att* site due to poor spatial resolution (Fig. S1 *C*). Therefore, the kinetics information presented in this work is limited to the formation of PS complexes (two integrase dimers bind to two *att* sites) through to completion of recombination. The dwell-time histograms for the PS and NP states of the *att*B-*att*P substrate were fit to a single-exponential algorithm to obtain the formation rate constants for PS and NP complexes (Fig. 4).

**Table 1:** Kinetics of recombination between *att* sites mediated by *LI* integrase or mutant *LI* integrase and kinetics of recombination between *att* sites mediated by *wt φC31* integrase or ΔCC *φC31* integrase

Reaction conditions			*k*-NP_f_ (10^4^ M^−1^ s^−1^)	*k*-PS_f_ (10^4^ M^−1^ s^−1^)	*k*-RS_f_ (10^−1^ s^−1^)	*k*-WS_f_ (10^−1^ s^−1^)	*k*-WS_d_ (10^−1^ s^−1^)	*k*-NP_d_ (10^−2^ s^−1^)	*k*-PS_d_ (10^−1^ s^−1^)
*attB-attP*	*LI* integrase	*wt*	1.8 ± 0.1	1.8 ± 0.1	12 ± 1	11 ± 1	1.1 ± 0.1	5.2 ± 0.5	12 ± 1
		S10A	3.1 ± 0.4	2.2 ± 0.1	ND	9.1 ± 0.5	1.6 ± 0.1	5.6 ± 0.3	11 ± 1
		K362A	1.8 ± 0.2	1.1 ± 0.1	9.5 ± 1.1	10 ± 0._1_	1.2 ± 0.1	2.9 ± 0.3	13 ± 1
		ΔCC	0.56 ± 0.15	0.46 ± 0.03	>15^a^	6. 5 ± 0.6	1.5 ± 0.2	0.54 ± 0.06	14 ± 0
	*φC31* integrase	*wt*	6.2 ± 1.1	9.1 ± 0.4	2.2 ± 0.3	3.2 ± 0.2	0.45 ± 0.02	2.2 ± 0.2	8.3 ± 0.7
		ΔCC	6.5 ± 0.8	11 ± 1	ND	1.7 ± 0.0	0.34 ± 0.02	1.1 ± 0.1	7.9 ± 0.9
		E449K^b^	8.8 ± 1.0	8.4 ± 0. 5	1.7 ± 0.4	2.3 ± 0.5	0.98 ± 0.05	3.6 ± 0.3	9.2 ± 1.2
*attL-attR*	*LI* integrase	*wt*	0.80 ± 0.10	0.89 ± 0.07	ND	3.8 ± 0.6	1.0 ± 0.1	5.8 ± 0.4	5.4 ± 0.8
		S10A	0.83 ± 0.13	1.4 ± 0.1	ND	3.5 ± 0.5	0.91 ± 0.04	5.4 ± 0.4	4.1 ± 0.4
		K362A	0.37 ± 0.02	0.34 ± 0.02	>15^a^	10 ± 1	1.1 ± 0.1	2.5 ± 0.2	9.7 ± 1.3
		ΔCC	0.27 ± 0.02	0.31 ± 0.05	>15^a^	7.8 ± 0.5	1.5 ± 0.1	1.1 ± 0.2	12 ± 0
	*φC31* integrase	*wt*	7.7 ± 1.4	10 ± 1	ND	0.40 ± 0.04	0.15 ± 0.02	0.84 ± 0.14	6.7 ± 0.4
		ΔCC	8.9 ± 0.5	8.7 ± 0.8	ND	0.13 ± 0.02	0.090 ± 0.010	0.57 ± 0.09	9.2 ± 0.6
		E449K^b^	4.9 ± 0.7	5.9 ± 1.0	2.4 ± 0.1	0.49 ± 0.04	0.59 ± 0.03	3.2 ± 0.2	5.5 ± 0.4

The rate constants were determined by fitting the dwell times to a single-exponential model. ND, not determined.

Comparable PS complex formation rates were obtained for *LI* Int^S10A^ in comparison to *wt LI* Int (Fig. 4 *C*). However, the estimated rate of PS complex formation (*k*-PS_f_) for *LI* Int^K362^ and *LI* Int ΔCC were 1.6- to 5-fold lower than that for *wt LI* integrase (Figs. 4 *C* and S3; Table 1). This result indicates that defects in CC interactions have a detrimental effect on the initial binding of *att* sites by the Int dimer to form both PS and NP complexes. The kinetic analysis of the NP complexes gave a ∼2- to 5-fold lower rate of complex dissociation (*k*-NP_d_) for *LI* Int^K362^ and *LI* Int ΔCC than for *wt LI* integrase (Fig. 5). The lower rate of dissociation for NP complexes is also likely to reduce the opportunities for a recombination-competent synapse to form, leading to a lower recombination efficiency.

A similar kinetic analysis was applied to the attL-attR substrate. A ∼3-fold lower *k*-WS_f_ for *wt LI* integrase and *LI* Int^S10A^ were obtained in comparison to those on the attB-attP substrate, resulting in a reduced synapsis efficiency (from ∼93.3% to 66.0% for *wt LI* integrase and from ∼94.5% to 77.8% for *LI* Int^S10A^) (Fig. S3). Moreover, a ∼2-fold lower *k*-PS_f_ for *wt LI* integrase and *LI* Int^S10A^ (Fig. 4 *D*) and a ∼3-fold lower *k*-PS_f_ for *LI* Int^K362^ (Fig. 4 *D*) were obtained in comparison to those on *attB-attP* substrate (Fig. 4 *C*), resulting in reduced recombination activities (Fig. 3 *B*). On the contrary, a comparable *k*-PS_f_ was obtained for *LI* Int ΔCC on *att*L-*att*R and *attB-attP* substrates (Fig. 4 *D*), consistent with no significant change in determined bound fraction (∼16.1% on *att*L-*att*R vs. ∼17.8% on *attB-attP*) (Fig. 3). Moreover, a ∼2-fold increased *k*-NP_d_ was obtained for *LI* Int ΔCC, indicating an accelerating dissociation of a nonproductively occupied *att*L-*att*R, thus affording it the chance to reattempt synapsis. Both of these properties allow *LI* Int ΔCC to maintain a detectable final recombination efficiency of 2.2% on attL-attR (Fig. 3 *B*). For another serine recombinase, φC31 Int, our previous TPM and fluorescence correlation spectroscopy experimental results indicated that faster dissociation of aberrant WS complexes and NP complexes can kinetically promote the reassembly of recombination-competent synaptic complexes (RS complexes), thus enhancing the recombination efficiency (28). For *LI* integrase, we found that the PS complex formation rate for *LI* Int ΔCC is unaffected, but an accelerated dissociation of NP complexes was found, contributing to an enhanced recombination efficiency on *att*L-*att*R substrates. Moreover, the dissociation rate constant of WS complexes (*k*-WS_d_) for *LI* integrase is ∼3- to 5-fold larger than that of φC31 integrase, suggesting that the dissociation of WS complexes will not impede the reassembly of recombination-competent RS complexes (Fig. S4 and Table 1) (28).

Differences in synaptic architecture can cause a subtle change in the effective length of DNA. Our earlier analyses of tyrosine and serine recombinases showed that the entry and exit points of DNA with respect to the synapsed sites can result in a subtle but detectable change in the BM amplitude of the attached bead. The tethered DNA behaves as if it were slightly shorter when the entry and exit points are at the same end of the synapse (toward the coverslip-glass surface) in comparison to when they are at opposite ends of the synapse. Therefore, synaptic states with a parallel-like alignment exhibit higher BM amplitude for DNA substrates containing *att* sites oriented head-to-tail. On the contrary, a lower BM amplitude is observed for synapse states with anti-parallel-like alignment (25,26,28,35). In our previous study, we showed that there are two different synaptic conformations generated by φC31 integrase that can be distinguished by their BM amplitudes (28). The lower BM amplitude distribution represents incorrectly oriented sites within the synapse (presumably in an anti-parallel-like configuration), and the higher BM amplitude distribution represents correct synaptic conformations (presumably parallel-like alignment) (28). Interestingly, BM amplitudes of both RS complexes and WS complexes generated by *wt LI* integrases and mutant *LI* integrases exhibited a single-Gaussian distribution, suggesting the exclusive presence of properly oriented *att* sites (parallel-like) in synapsis, even when the synapse that harbors these sites is nonfunctional (Fig. S6 and Table 2).

**Table 2:** BM amplitudes of *attB-attP* and *attL-attR* synaptic complexes, recombination-proficient (RS) and recombination-blocked (WS) complexes by *wt LI* integrases or mutant *LI* integrases, and by *wt φC31* integrase or ΔCC *φC31* integrase

Substrate	Proteins		BM amplitude of synapse (nm)	
			Recombination-proficient	Recombination-blocked
*attB-attP* (parallel)	*LI* integrase	*wt*	40.3 ± 3.1	39.2 ± 5.3
		SA10A		40.1 ± 7.5
		K362	39.5 ± 3.5	39.±4.5
		ΔCC	43.9 ± 3.9	41.5 ± 6.0
	*φC31* integrase	*wt*	49.3 ± 2.9	51.8 ± 8.8
		ΔCC	ND	35.0 ± 3.3 (44.6%)
				48.9 ± 3.3 (55.4%)
*attL-attR* (parallel)	*LI* integrase	*wt*	ND	43.6 ± 6.3
		SA10A	ND	40.5 ± 8.1
		K362	41.3 ± 4.5	40.1 ± 4.7
		ΔCC	43.6 ± 4.0	41.0 ± 6.7
	*φC31* integrase	*wt*	ND	41.9 ± 2.0 (24.6%)
				52.6 ± 3.6 (75.4%)
		ΔCC	ND	38.3 ± 8.5 (68.0%)
				51.9 ± 2.9 (34.0%)
*attB-attP* (inverse)	*LI* integrase	*wt*		40.1 ± 7.9
		SA10A		39.7 ± 5.6
		K362		39.5 ± 6.9
		ΔCC		39.0 ± 7.0
	*φC31* integrase	*wt*		37.5 ± 3.0 (43.7%)
				46.7 ± 3.3 (56.3%)
		ΔCC		35.1 ± 6.2 (36.7%)
				48.5 ± 3.2 (63.3%)

The BM amplitude histograms are shown in Figs. S7 and S8. The parallel-like (correct, high BM) and anti-parallel-like (incorrect, low BM) synaptic geometries for DNA substrates with two *att* sites in head-to-tail orientation are schematically illustrated in the previous report (30).

### Deletion of the φC31 integrase coiled coil abolishes recombination activity

**Figure 6:**
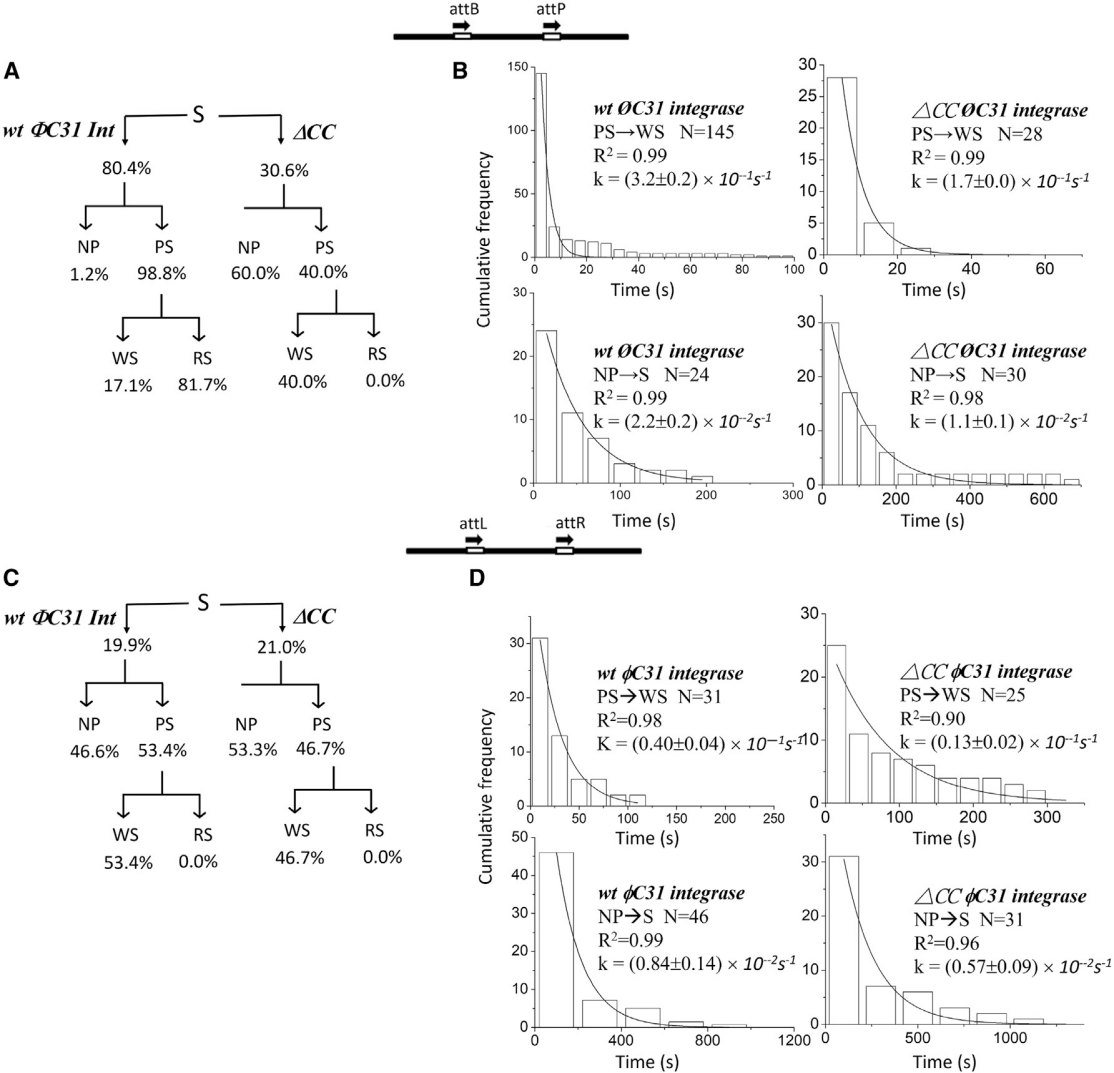
Kinetic analysis of φC31 integrase wayward synaptic (WS) complex association and nonproductive (NP) complex dissociation. (*A*) The reaction behaviors of *wt φC31* integrases and ΔCC *φC31* integrases on DNA molecules with *attB × attP* sites in head-to-tail orientation. (*B*) Histograms showing the *attB × attP* WS complex association rate constants and NP complex dissociation rate constants obtained for *wt* φC31 integrase and ΔCC φC31 integrase. (*C*) The reaction behaviors of *wt φC31* integrases and ΔCC *φC31* integrases on DNA molecules with *attL × attR* sites in head-to-tail orientation. (*D*) Histograms showing the *attL × attR* WS complex association rate constants and NP complex dissociation rate constants obtained for *wt φC31* integrases and ΔCC *φC31* integrases. The data were fitted to a single-exponential decay algorithm. *N* annotated on each graph is the number of molecules observed. All data were fitted by using 8.0 Origin. The error is in 95.5% CI.

In our previous studies, we reported that φC31 Int^E449K^ hastens the disassembly of futile PS complexes and synaptic complexes and thermodynamically promotes the formation of recombination-competent states leading to higher recombination efficiency on *attB* × *attP* and *attL* × *attR* in comparison to *wt* φC31 integrase (28). Here, similar TPM experiments and analysis procedures as those described for *LI* Int were applied to a CC domain deletion of φC31 integrase (ΔCC φC31 Int) (Fig. S7). The ΔCC φC31 Int bound only 30.6% of *attB* × *attP* DNA substrate, compared to 80.4% bound by *wt* φC31 Int (Fig. 6 *A*). Of the fraction bound by ΔCC φC31 Int, ∼60% were unable to successfully synapse, and none of the synaptic complexes proceeded to successful recombination (Fig. 6 *A*). Similar to ΔCC *LI* Int, a 2-fold lower *k*-NP_d_ was observed for ΔCC φC31 Int than for *wt* φC31 Int, indicating that higher numbers of PS molecules were trapped in NP complexes preventing successful recombination (Fig. 6). Unlike *LI* Int, comparable association rate constants (*k*-PS_f_ and *k*-NP_f_) were obtained for ΔCC φC31 Int and *wt* φC31 Int, indicating that the deletion of the coiled coil does not significantly affect the first step of the recombination process, i.e., the binding of integrase dimers to *att* sites (Table 1 and Fig. S7). On the contrary, a 1.7-fold lower *k*-WS_f_ was obtained for ΔCC φC31 Int vs. *wt* φC31 Int, highlighting the importance of the CC domain for synapse formation.

In our previous studies, it has been pointed out that the topological difference in synapse complexes can result in a detectable difference in BM amplitude of WS observed for φC31 Int (26,28). Here, BM amplitudes of WS complexes generated by *wt* φC31 Int exhibited a unimodal distribution, suggesting the preferable formation of parallel-like *att*-site orientation, even in those synaptic structures that do not successfully execute recombination, consistent with previous reports (Fig. S8) (26,28). However, the broad distribution of the WS complexes formed by *wt* φC31 Int cannot rule out the possibility of the presence of both correctly and incorrectly oriented sites (parallel-like and anti-parallel-like) in the inactive synaptic structures. On the contrary, the WS complexes formed by ΔCC φC31 Int exhibited a bimodal distribution with mean BM amplitudes of 48.9 ± 3.3 nm (55.4%) and 35.0 ± 3.3 nm (44.6%), suggesting they are populated by analogous inactive conformations that include both parallel-like and anti-parallel-like *att*-site arrangements (Fig. S8).

It should be noted that we used different reaction buffers for the respective *LI* Int and φC31 Int experiments. The *LI* Int buffer has a higher ionic strength compared to the φC31 buffer (150 mM KCl vs. 100 mM NaCl) and includes 5 mM MgCl_2_. These two buffer compositions match those used for the majority of published molecular and biochemical characterization of the two integrases (12,18,20,24) and, therefore, our data should be comparable with previous work. To test whether buffer conditions affect the kinetics of φC31 activity, we repeated the TPM experiments for *wt* φC31 Int and ΔCC φC31 Int in the *LI* Int buffer on the DNA substrates containing *attB* × *attP* in head-to-tail orientation. These data demonstrate that buffer composition does not significantly alter the distribution of BM amplitudes (Fig. S9), indicating that *att-*site orientation in Int-DNA complexes is unaffected. Meanwhile, the higher ionic strength did lead to an approximately 2- to 3-fold slower synapse-formation rate (i.e., *k*-RS_f_ and *k*-WS_f_ became smaller) and a faster synapse-decomposition rate (i.e., *k*-WS_d_ became slightly larger) (Table S3). The association and dissociation rate constants of synaptic complexes for φC31 Int in *LI* Int buffer are still smaller than those for *LI* Int in *LI* Int buffer and, crucially, the effects of *LI* buffer on *wt* φC31 Int and ΔCC φC31 Int activity are roughly equivalent.

Similar experiments were performed for *wt* φC31 Int and ΔCC φC31 Int with DNA containing *attL × attR* in head-to-tail orientation. The *wt* and ΔCC proteins produced comparable DNA-binding activities of 19.9% and 21.0%, respectively, both of which were substantially lower than for the corresponding *attB* × *attP* reactions (Fig. 6 *C*). Our previous work with *wt* φC31 Int on *attL × attR* revealed that decreased dissociation rate constants (*k*-WS_d_ and *k*-NP_d_) kinetically suppressed the probability of successful recombination in the absence of RDF, gp3 (28). Similar behavior was observed here for ΔCC φC31 Int (Fig. 6 *D* and Table 1); however, a 4-fold lower association rate constant, *k*-WS_f_, was obtained for ΔCC φC31 Int than for *wt*. These data support the hypothesis that the CC motif plays an important role in synapse formation. Moreover, the distributions of the WS complexes formed by both *wt* φC31 Int and ΔCC φC31 Int were bimodal with mean amplitudes of ∼38.3–41.9 nm, representing a recombination-incompetent anti-parallel-like *att-*site arrangement, and ∼51.9–52.6 nm, representing a recombination-competent parallel-like *att-*site arrangement (Fig. S8). However, a higher proportion (68.0%) of WS complexes formed by ΔCC φC31 Int exhibit a lower BM amplitude in comparison to *wt* φC31 Int (24.6%), suggesting that deletion of the CC domain leads to a defect in proper orientation of *attL* × *attR*, even though the synapse that harbors these sites is recombination incompetent.

## Discussion

### Thermodynamic aspects of integrase recombination

The data presented in this study provide important insights into the process of recombination by two model serine integrases in the absence of RDF, *LI* Int and φC31 Int, and the role of the conserved C-terminal CC motif. The CC motif has previously been shown to play an important role in recombination, but here we explore the thermodynamics at the single-molecule level. CC deletion mutants of both integrases showed a remarkable reduction in *attB-attP* DNA binding in comparison to *wt* integrase (Figs. 2 and 6). Moreover, the ΔCC Int-DNA complexes are predominantly biased toward nonproductive forms (NP) and against productive synaptic complexes. These data are consistent with previous work showing that deletion of the CC motif impairs Int dimer-dimer interactions that are essential for recombination, leading to a lower recombination efficiency or even an abolished activity (12,18,19,20,24).

In the absence of gp3, we observed a marked reduction in the yield of wt *LI* and φC31 integrase PS complexes on *attL*-*attR* DNA substrates in comparison to experiments with *attB-attP* DNA (Figs. 3 and 6), consistent with the notion that formation of the synapse is a critical regulatory step in determining the directionality of recombination (21,36). However, the ΔCC *LI* Int and ΔCC φC31 Int proteins had relatively small difference in the fraction of *att* sites bound, the fraction of PS complex formation, or overall recombination efficiency for *attL* × *attR* substrates in comparison to *attB* × *attP* substrates (Figs. 2 and 6), indicating that, consistent with previous models, deletion of the CC motif obliterates its regulatory function (18). Interestingly, ΔCC *LI* Int maintained similar overall reaction activities of ∼1.6% and ∼2.2% on *attB* × *attP* and *attL* × *attR* substrates, respectively, but ΔCC φC31 Int was totally inactive on both substrates (Fig. 6). These data suggest that the regulatory role of the CC motifs is likely to be different in these two-serine recombinase families and will require further investigation.

### Kinetic aspects of integrase recombination

Although current experimental results were obtained at room temperature (22°C ± 1°C), the information obtained can be more precisely close to physiological condition by implementation of a precise temperature-controlling system in the future. Based on current experimental results obtained, the derivative K362A and ΔCC mutants have a decreased rate of PS complex formation (*k*-PS_f_) with both *attB × attP* and *attL × attR* substrates in comparison to *wt LI* integrase (Table 1). In contrast, there was only a slight difference in *k*-PS_f_ rates for ΔCC φC31 Int compared to *wt* φC31 Int with both *att* substrate pairs (Table 1). Due to the spatial-resolution limitation of our TPM system, the *k*-PS_f_ was determined by the detectable decrease in BM amplitude representing the formation of PS complexes, i.e., binding of two integrase dimers on both *att* sites.

It is believed that *wt LI* integrase binds to the *att* site as a dimer (18). The *LI* Int monomer-dimer *K*_D_ is estimated to be 32 nM, which is lower than the experimental concentration of 200 nM used in this project, and thus the *wt* protein is expected to be in the dimeric form in our experiments (18). Importantly, the CC motifs stabilize dimers formed by *LI* Int NTD interactions, and a ∼100-fold weaker monomer-dimer *K*_D_ was reported for mutants that interfere with coiled-coil interactions (18). Therefore, *LI* integrase with a defective dimerization interface (*LI* Int^K362^ and ΔCC *LI* Int) tends to be in the monomer form. It took longer to observe PS complexes formed by sequential binding of integrase monomers on both *att* sites for ΔCC *LI* Int in comparison to *wt LI* Int, leading to a 5-fold decreased *k*-PS_f_. Meanwhile, the *k*-PS_f_ of *wt LI* Int was ∼2-fold lower for *attL × attR* than *attB-attP*, but the *k*-PS_f_ for ΔCC *LI* Int was comparable for both *attB × attP* and *attL × attR* substrates. This kinetic behavior supports our observation that the fraction of bound molecules formed by *wt LI* Int decreased from 81.1% *attB × attP* to 48.6% *attL × attR*, but a similar bound fraction was observed for ΔCC *LI* Int on both substrates.

For φC31 integrase, it has been suggested that the putative CC motif is either buried or sequestered when full-length φC31 integrase is free in solution, and the coiled coil is only free to mediate protein-protein interactions when Int is bound to DNA (24). Moreover, it has been reported that the CTD alone cannot dimerize in solution but full-length φC31 integrase can, via the NTD interactions (24). In our hands the ΔCC φC31 Int can dimerize in solution and bind to all *att*-site DNA in a manner comparable to that of the wt (Fig. S10), which corroborates our observation of no change in *k*-PS_f_ for *wt* φC31 Int vs. ΔCC φC31 Int (Table 1). Furthermore, the association constant of φC31 Int exhibited no *att-*site dependence. Notably, the fraction of *attL × attR* DNA bound by *wt* φC31 Int is nearly 4-fold lower than for *attB × attP* (80.4% vs. 19.9%), which suggests that there are other unknown factors that affect the observed bound fraction beyond DNA-binding affinity alone.

Our TPM analyses also suggest that the influence of CC motifs on association of nonfunctional synaptic complexes (PS → WS) and the dissociation of nonfunctional presynaptic complexes (NP → S) are similar for both *LI* Int and φC31 Int (Figs. 5 and 6; Table 1), both of which increase the yield of successful recombination products within the timescale of the assays. Fast association rates enhance production of PS complexes available for recombination, while rapid dissociation of inactive NP complexes allows the reformation of PS complexes and subsequent recombination. In contrast, ΔCC *LI* Int and ΔCC φC31 Int produced an ∼1.7-fold smaller association rate of nonfunctional wayward synaptic complexes (*k*-WS_f_) and at least 2-fold smaller dissociation rate constant of NP complexes (*k*-NP_d_) compared to the respective *wt* integrases (Table 1). Both factors contributed to an accumulation of inactive PS complexes, i.e., ∼50% of observed Int-DNA complexes were in the inactive form (NP) (Figs. 3 and 6), which in turn led to decreased or abolished recombination efficiency (Figs. 3 and 6). It has been proposed that the CC motifs in *LI* Int serve an auxiliary role to fortify CTD and NTD interactions (18). Based on our TPM results, deletion of the CC motifs almost eliminates the thermodynamic and kinetic differences between the *attB × attP* and *attL × attR* reactions. These data strongly support the previously proposed CC motif interaction regulatory model for *LI* Int (18). In contrast, previous data for φC31 Int demonstrated that the CC motifs are crucial and prerequisite for CTD interactions that are known to be important for the formation of the NTD synaptic interface (24). Here we show that deletion of the CC motif totally abolishes the recombination ability of φC31 Int with both *attB* × *attP* and *attL × attR*, which is likely to be due to severe deficiencies or perhaps elimination of DNA-dependent CTD interactions and consequent impairment of catalytic NTD interactions.

### Conformational differences between *Listeria innocua* integrase and φC31 integrase in synaptic complexes

The mean BM amplitude of RS complexes formed by *wt LI* integrase and its mutants with *attB*-*attP* or *attL-attR* DNA was ∼40.0–42.8 nm (Fig. S6 and Table 2), which is thought to correspond to *att* sites aligned in parallel (25,26,28,35). Inactive WS complexes that synapse but do not produce excision products exhibited similar BM amplitude of 39.2–43.6 nm. Moreover, synaptic complexes form by Int bound to *attB*-*attP* sites in head-to-head orientation, including RS complexes that are presumably aligned with parallel-like geometry with exit end facing toward coverslip-glass surface and WS complexes, also exhibited similar BM amplitudes of ∼39.0–40.1 nm (Fig. S6 and Table 2). These observations suggest that the differences in synaptic architecture formed by *LI* integrase are too subtle to be discriminated by TPM experiments.

In contrast, complexes generated by *wt* φC31 Int and ΔCC φC31 Int exhibit obvious differences in their synaptic conformations (Fig. S8). It has been reported that a high amplitude distribution (∼46.7–52.6 nm) is indicative of a parallel-like synaptic conformation, while a low amplitude distribution (∼35.0–41.9 nm) represents a nonproductive anti-parallel-like conformation for *wt* φC31 (26,28). WS complexes formed by *wt* φC31 Int with *attB*-*attP* produced a single high amplitude distribution, akin to productive RS complexes. WS complexes comprising *wt* φC31 Int and *attL* × *attR* produced a bimodal BM amplitude distribution, most of which exhibited a high BM amplitude (75.4%). In contrast, ΔCC φC31 Int produced WS complexes with bimodal BM amplitude distributions when bound to both *attB* × *attP* and *attL* × *attR*. Moreover, a larger proportion of the complexes exhibited low BM amplitude (44.6% on *attB* × *attP*, 68.0% on *attL* × *attR*), indicating that *att-*site alignment during synapsis is prelocked in an anti-parallel-like orientation and is thus biased against successful formation of recombinant products. These data indicate that the lack of a CC domain not only severely inhibits φC31 Int recombination but also causes a defect in proper orientation of the *att* sites during synapsis. These two factors are likely to be the cause of abolished recombination activity observed for ΔCC φC31 integrase (Figs. 6 and S8) (20).

It has been proposed that the RDF for *Listeria* phage A118 and φC31 phage binds to the base of the CC motif, perhaps altering the trajectory of the motif to facilitate the formation of a synaptic complex (19,20,37). The present TPM analysis of the *Listeria innocua* integrase and φC31 integrase systems has revealed kinetic and thermodynamic similarities and differences that provide important insights into the contribution of the CC domains to the respective recombination behaviors. Our TPM experiments suggest that the CC motifs in *LI* Int serve an auxiliary role to fortify synaptic interface interactions. In contrast, φC31 Int CC motifs are an essential prerequisite for synaptic interface. In our previous studies on φC31 integrase, we showed that the φC31 RDF promotes formation of synapses with proper *att*-site orientation (parallel-like) and promotes faster dissociation of nonproductively bound Int from *attL* × *attR* synapses (26,28). Here we observed >3-fold faster dissociation of abortive synapses (wayward complexes) for *LI* integrase without the aid of RDF on both *attB* × *attP* and *attL* × *attR* substrates in comparison to φC31 integrase, suggesting the presence of different reaction behaviors for these two serine integrases. Overall, our data suggest that the mechanism of RDF regulation of recombination could be different for *LI* Int and φC31 Int, and more work is required to unveil the precise differences between different serine integrases.

## Author contributions

Y.-W.C. and B.-Y.S. contributed to performing purification of φC31 integrase and related mutants and conducted single-molecule experiments and data analysis. G.D.V.D. contributed to performing purification of wt LI integrase and related mutants. P.F. contributed to electrophoretic mobility shift assay and manuscript writing. H.-F.F. contributed to the design of the study, single-molecule experiments, independent analysis, and manuscript writing and presentation.

## Supporting material

Document S1. Figures S1–S12 and Tables S1–S3

Document S2. Article plus supporting material
