# Supplementary material for: The influence of coiled-coil motif of serine recombinase toward the directionality regulation
Source: Biophys J. 2023 Nov 16;122(24):4656–69. doi: 10.1016/j.bpj.2023.11.009 (PMC10754689; doi:10.1016/j.bpj.2023.11.009)
Supplement: Document S1. Figures S1–S12 and Tables S1–S3 [file mmc1.pdf]

Biophysical Journal, Volume *122*

## Supplemental information

# The influence of coiled-coil motif of serine recombinase toward the directionality regulation

**Yei-Wei Chen, Bo-Yu Su, Gregory D. Van Duyne, Paul Fogg, and Hsiu-Fang Fan**

# SUPPLEMENTARY DATA

## The influence of coiled-coil motif of *serine recombinase* toward the directionality regulation

**Yei-Wei Chen [1,2.3+], Bo-Yu Su[4+], Gregory D Van Duyne[5], Paul Fogg [6*], Hsiu-Fang Fan[1,2.3*]**

[1] Institute of Medical Science and Technology, National Sun Yat-sen University, Taiwan

[2] Department of Chemistry, National Sun Yat-sen University, Taiwan

[3] Aerosol Science Research Center, National Sun Yat-sen University, Taiwan

[4] Department of Life Sciences and Institute of Genome Sciences, National Yang-Ming University, Taiwan

[5] Perelman School of Medicine, University of Pennsylvania, USA

[6] Biology Department and York Biomedical Research Institute (YBRI), University of York, York, United Kingdom

+: These authors have equal contributions to this work

* Corresponding author

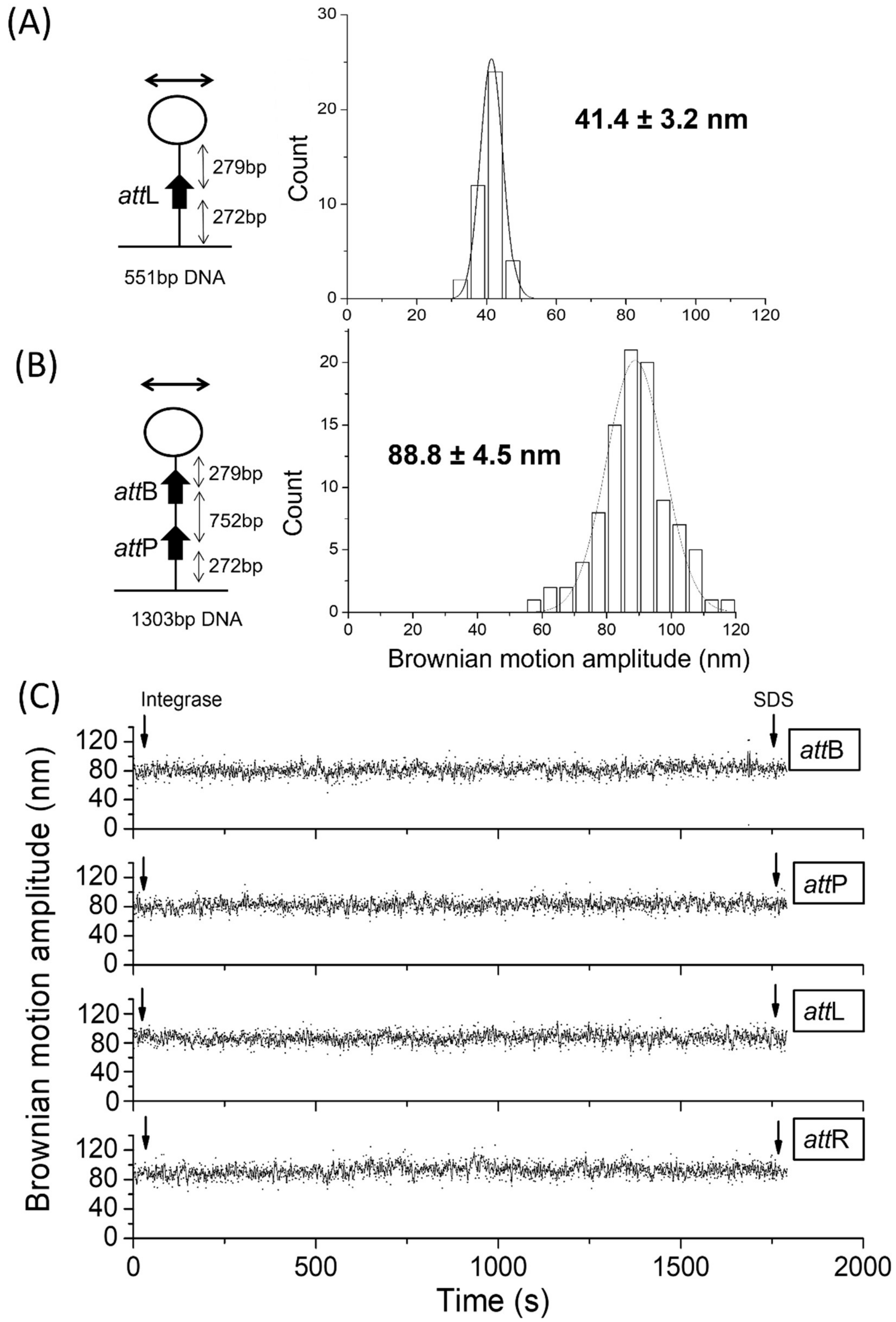


**Supplemental Figure 1** *The Brownian motion amplitude histogram of LI integrase excision product DNA (551bp) and substrate DNA (1303bp).* (**A**). Schematic illustration of 551 bp product DNA molecules containing *attL*, and the Brownian motion amplitude

with an average value of 41.4 ± 3.2 nm. (**B**)**.** Schematic illustration of *1303bp substrate DNA molecules* containing *attB-attP* in head-to tail orientations, and the Brownian motion amplitude with an average value of 88.5 ± 4.5 nm. All the data were fitted using Origin 8.0. The error is in 95.5% CL. (C)**.** The representative time traces illustrate 1303 bp DNA molecules containing single *att* sites in response to the addition of *wt* LI integrase protein.

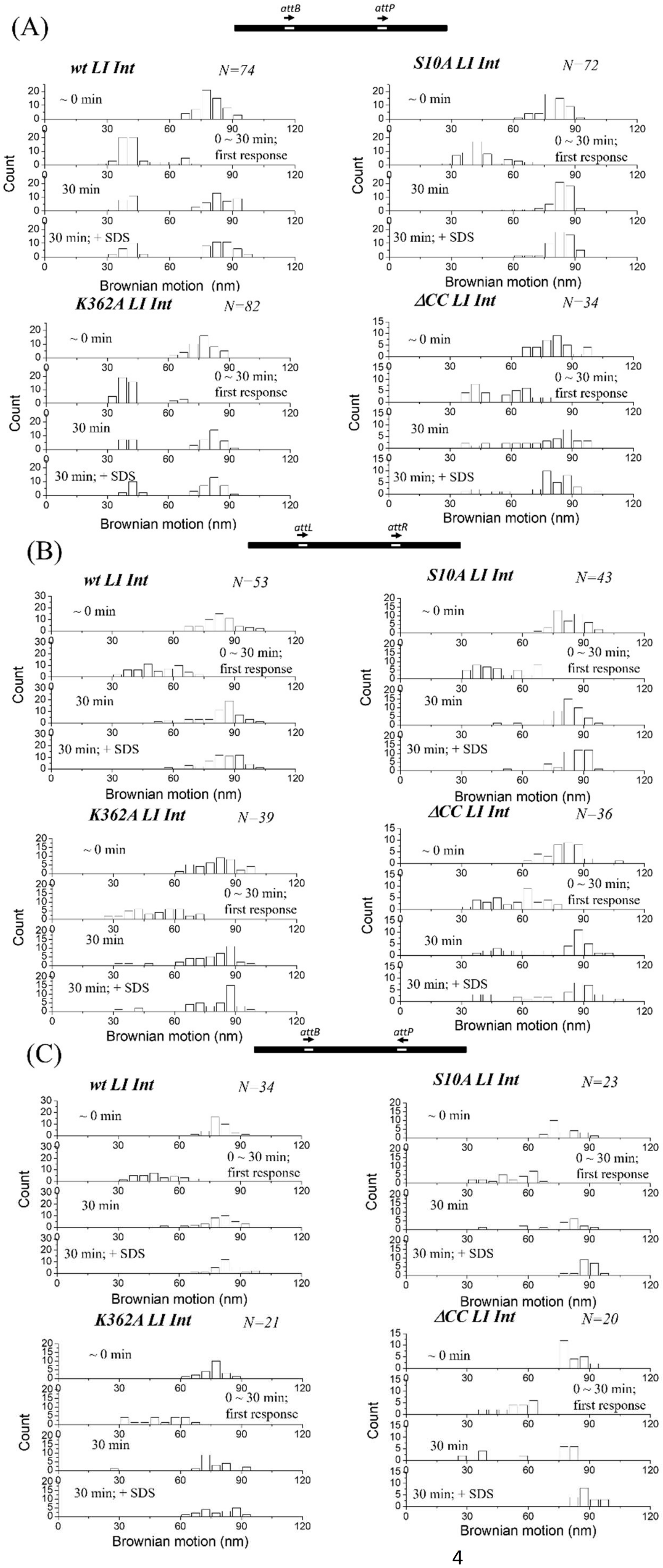
(A)
attB
attP
wt LI Int
N=74
S10A LI Int
N=72
K362A LI Int
N=82
ΔCC LI Int
N=34
(B)
attL
attR
wt LI Int
N=53
S10A LI Int
N=43
K362A LI Int
N=39
ΔCC LI Int
N=36
(C)
attB
attP
wt LI Int
N=34
S10A LI Int
N=23
K362A LI Int
N=21
ΔCC LI Int
N=20
~ 0 min
0 ~ 30 min; first response
30 min
30 min; + SDS
Count
Brownian motion (nm)

**Supplemental Figure 2** *Characterization of recombination by integrase in a substrate containing att sites in head-to-tail or head-to-head orientation shown above*. (**A**) The BM amplitude distribution of DNA molecules with *att*B-*att*P sites in head-to-tail orientation, (**B**) *att*L-*att*R sites in head-to-tail and (**C**) *att*B-*att*P sites in head-to-head orientation in response to the addition of wt *LI* integrases, S10A *LI* integrases, K362A *LI* integrases or ΔCC *LI* integrases. From top to bottom: just prior to the addition of integrase (-0 min), first response, at 30 min of incubation (just prior to SDS addition) and after SDS challenge, respectively.

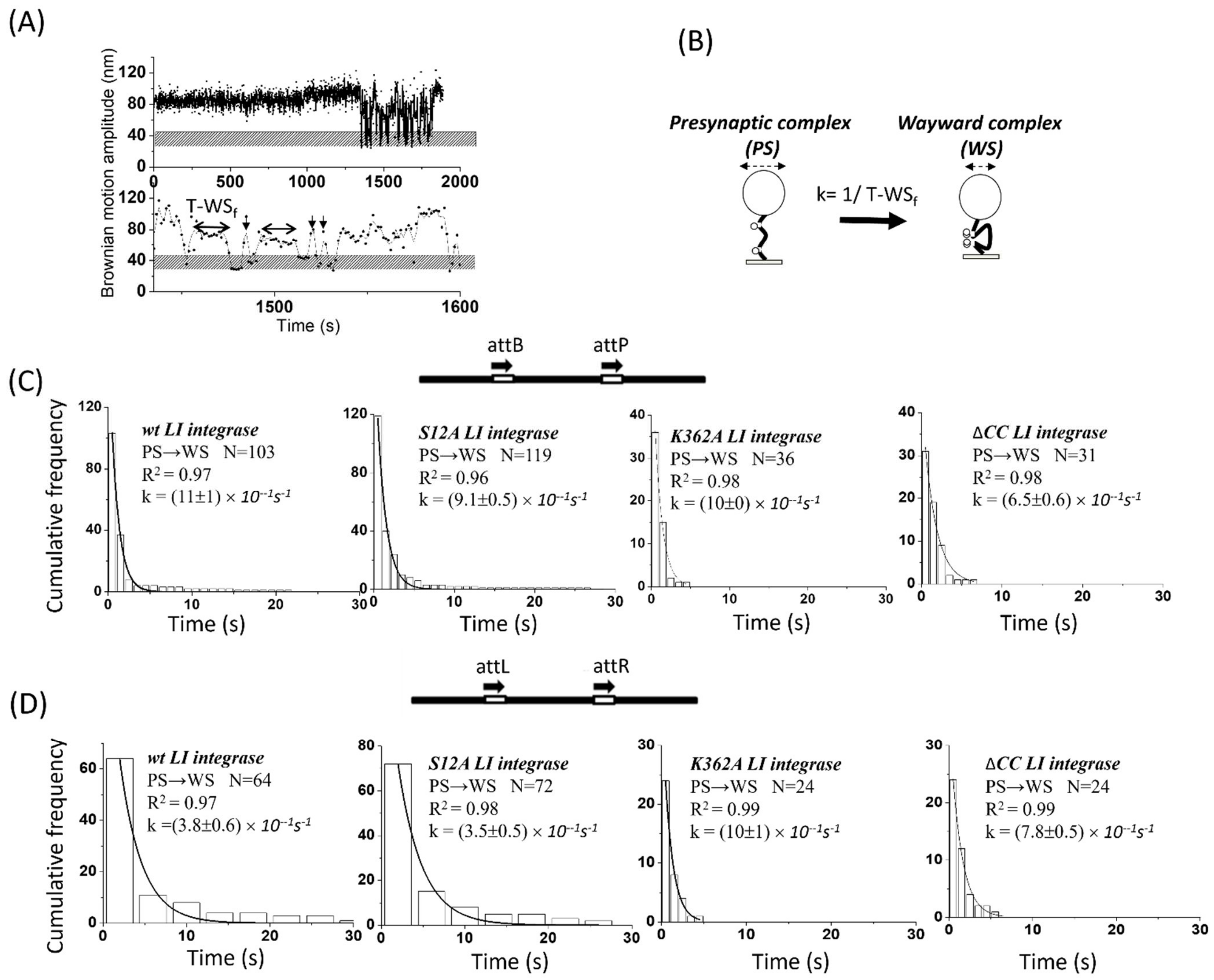
(A)
Brownian motion amplitude (nm)
T-WS_f
Time (s)
(B)
Presynaptic complex (PS)
Wayward complex (WS)
k= 1/ T-WS_f
(C)
attB
attP
Cumulative frequency
wt LI integrase
PS→WS N=103
R² = 0.97
k = (11±1) × 10⁻¹s⁻¹
S12A LI integrase
PS→WS N=119
R² = 0.96
k = (9.1±0.5) × 10⁻¹s⁻¹
K362A LI integrase
PS→WS N=36
R² = 0.98
k = (10±0) × 10⁻¹s⁻¹
ΔCC LI integrase
PS→WS N=31
R² = 0.98
k = (6.5±0.6) × 10⁻¹s⁻¹
Time (s)
(D)
attL
attR
Cumulative frequency
wt LI integrase
PS→WS N=64
R² = 0.97
k =(3.8±0.6) × 10⁻¹s⁻¹
S12A LI integrase
PS→WS N=72
R² = 0.98
k = (3.5±0.5) × 10⁻¹s⁻¹
K362A LI integrase
PS→WS N=24
R² = 0.99
k = (10±1) × 10⁻¹s⁻¹
ΔCC LI integrase
PS→WS N=24
R² = 0.99
k = (7.8±0.5) × 10⁻¹s⁻¹
Time (s)

**Supplemental Figure 3** *Kinetic analysis of 1303 bp DNA molecule containing attB-attP or attL-attR sites in head-to-tail orientation in response to LI integrases.* (**A**). The dwell times in the presynaptic state before synaptic complexes formation were pooled and fitted with a single exponential decay model (marked with double side arrows and downward arrows in the enlarged region). (**B**) Schematic illustration of integrase protein bound to substrate DNA in the TPM experimental set-up. (**C**) The *attB* x *attP* wayward complex association rate constants were obtained for wt *LI* integrases, S10A *LI* integrases, K362A *LI* integrases or ΔCC *LI* integrases respectively. (**D**) The *attL* x *attR* wayward complex association rate constants were obtained for wt *LI* integrases, S10A *LI* integrases, K362A *LI* integrases or ΔCC *LI* integrases respectively. The association rate constant was converted from the reciprocal of the association time with a unit of $s^{-1}$. The data were fitted to a single-exponential decay algorithm. The N mentioned above is the number of molecules observed. All the data were fitted using Origin 8.0. The error is in 95.5% CL.

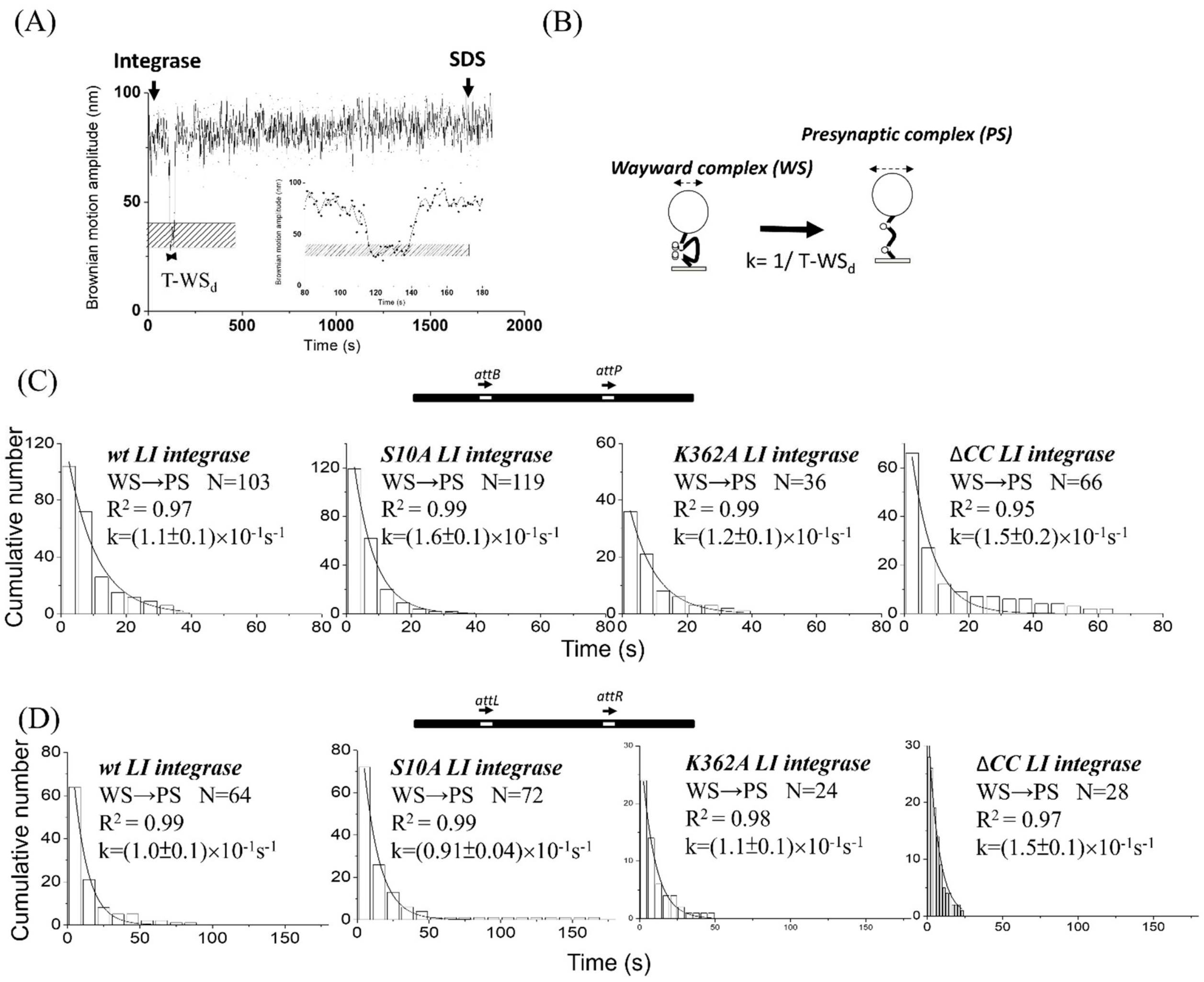

(A)
Integrase
SDS
Brownian motion amplitude (nm)
T-WSd
Time (s)
(B)
Presynaptic complex (PS)
Wayward complex (WS)
k= 1/ T-WSd
(C)
attB
attP
Cumulative number
wt LI integrase
WS→PS N=103
R2 = 0.97
k=(1.1±0.1)×10-1s-1
S10A LI integrase
WS→PS N=119
R2 = 0.99
k=(1.6±0.1)×10-1s-1
K362A LI integrase
WS→PS N=36
R2 = 0.99
k=(1.2±0.1)×10-1s-1
ΔCC LI integrase
WS→PS N=66
R2 = 0.95
k=(1.5±0.2)×10-1s-1
Time (s)
(D)
attL
attR
Cumulative number
wt LI integrase
WS→PS N=64
R2 = 0.99
k=(1.0±0.1)×10-1s-1
S10A LI integrase
WS→PS N=72
R2 = 0.99
k=(0.91±0.04)×10-1s-1
K362A LI integrase
WS→PS N=24
R2 = 0.98
k=(1.1±0.1)×10-1s-1
ΔCC LI integrase
WS→PS N=28
R2 = 0.97
k=(1.5±0.1)×10-1s-1
Time (s)

**Supplemental Figure 4** *Kinetic analysis of 1303 bp DNA molecule containing attB-attP or attL-attR sites in head-to-tail orientation in response to LI integrase.* **A**. The dwell times in the wayward complexes were pooled. **B.** The schematic illustration. **C.** The dissociation time of wayward complexes formed on *attB-attP* sites in head-to-tail orientation were obtained for wt *LI* integrases, S10A *LI* integrases, K362A *LI* integrases or ΔCC *LI* integrases. **D.** The dissociation time of wayward complexes formed on *attL-attR* sites in head-to-tail orientation were obtained for wt *LI* integrases, S10A *LI* integrases, K362A *LI* integrases or ΔCC *LI* integrases. The data were fitted to a single-exponential decay algorithm. The dissociation rate constant was converted from the reciprocal of the dissociation time with a unit of $s^{-1}$. The N mentioned above is the number of molecules observed. All the data were fitted using Origin 8.0. The error is in 95.5% CL. All fitting values are listed in Table I.

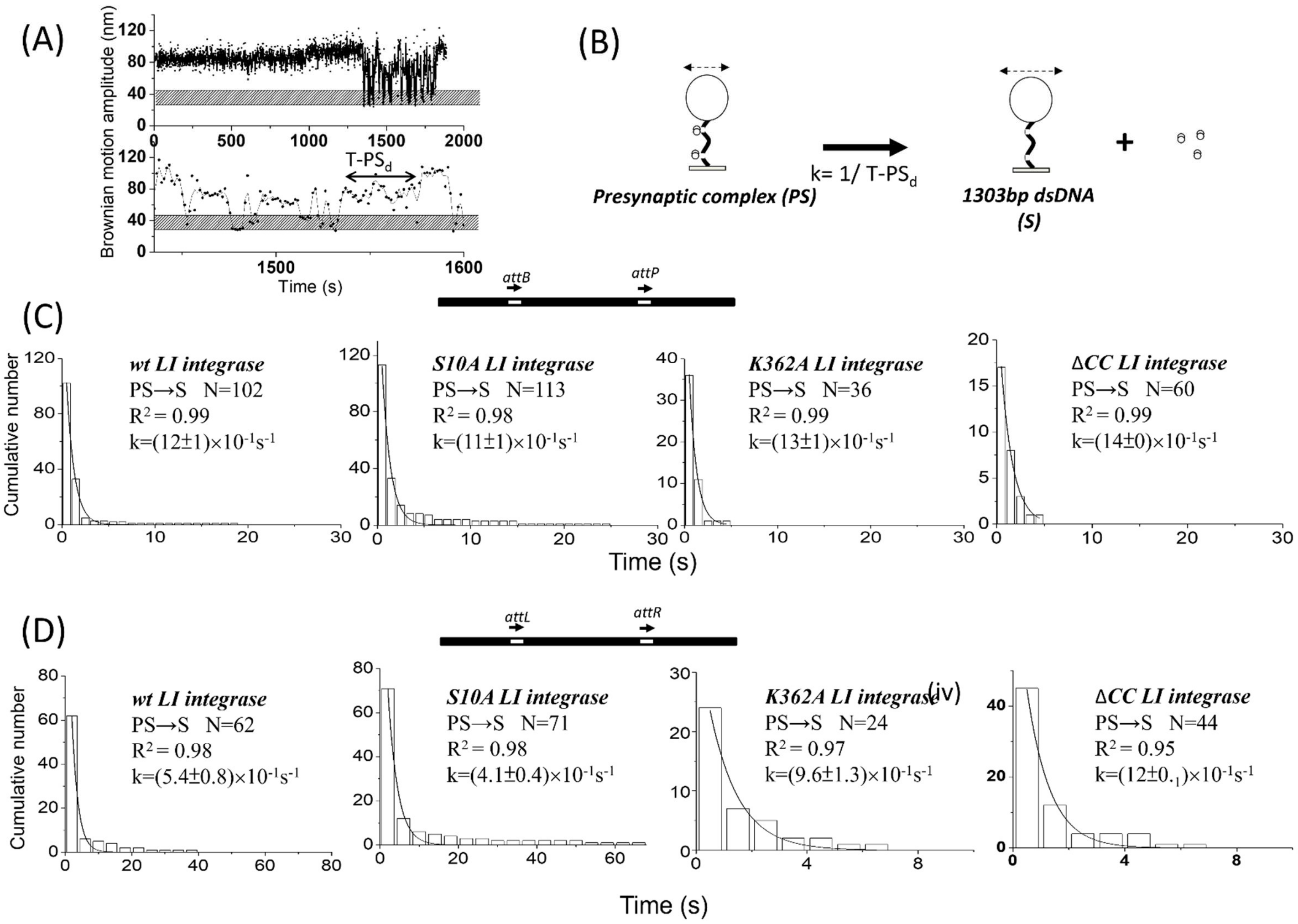
(A)
Brownian motion amplitude (nm)
T-PS$_d$
Time (s)
(B)
Presynaptic complex (PS)
k= 1/ T-PS$_d$
1303bp dsDNA (S)
attB
attP
(C)
Cumulative number
wt LI integrase
PS→S N=102
$R^2$ = 0.99
k=(12±1)×10$^{-1}$s$^{-1}$
S10A LI integrase
PS→S N=113
$R^2$ = 0.98
k=(11±1)×10$^{-1}$s$^{-1}$
K362A LI integrase
PS→S N=36
$R^2$ = 0.99
k=(13±1)×10$^{-1}$s$^{-1}$
ΔCC LI integrase
PS→S N=60
$R^2$ = 0.99
k=(14±0)×10$^{-1}$s$^{-1}$
Time (s)
(D)
attL
attR
Cumulative number
wt LI integrase
PS→S N=62
$R^2$ = 0.98
k=(5.4±0.8)×10$^{-1}$s$^{-1}$
S10A LI integrase
PS→S N=71
$R^2$ = 0.98
k=(4.1±0.4)×10$^{-1}$s$^{-1}$
K362A LI integrase (iv)
PS→S N=24
$R^2$ = 0.97
k=(9.6±1.3)×10$^{-1}$s$^{-1}$
ΔCC LI integrase
PS→S N=44
$R^2$ = 0.95
k=(12±0.$_1$)×10$^{-1}$s$^{-1}$
Time (s)

**Supplemental Figure 5** *Kinetic analysis of 1303 bp DNA molecule containing attB-attP or attL-attR sites in head-to-tail orientation in response to LI integrase.* **A**. The dwell times in the presynaptic complexes were pooled. **B.** The schematic illustration. **C.** The dissociation time of presynaptic complexes formed on *attB-attP* sites in head-to-tail orientation were obtained for wt *LI* integrases, S10A *LI* integrases, K362A *LI* integrases or ΔCC *LI* integrases, **D.** The dissociation time of presynaptic complexes formed on *attL-attR* sites in head-to-tail orientation were obtained for wt *LI* integrases, S10A *LI* integrases, K362A *LI* integrases or ΔCC *LI* integrases. The data were fitted to a single-exponential decay algorithm. The dissociation rate constant was converted from the reciprocal of the dissociation time with a unit of $s^{-1}$. The N mentioned above is the number of molecules observed. All the data were fitted using Origin 8.0. The error is in 95.5% CL. All fitting values are listed in Table I.

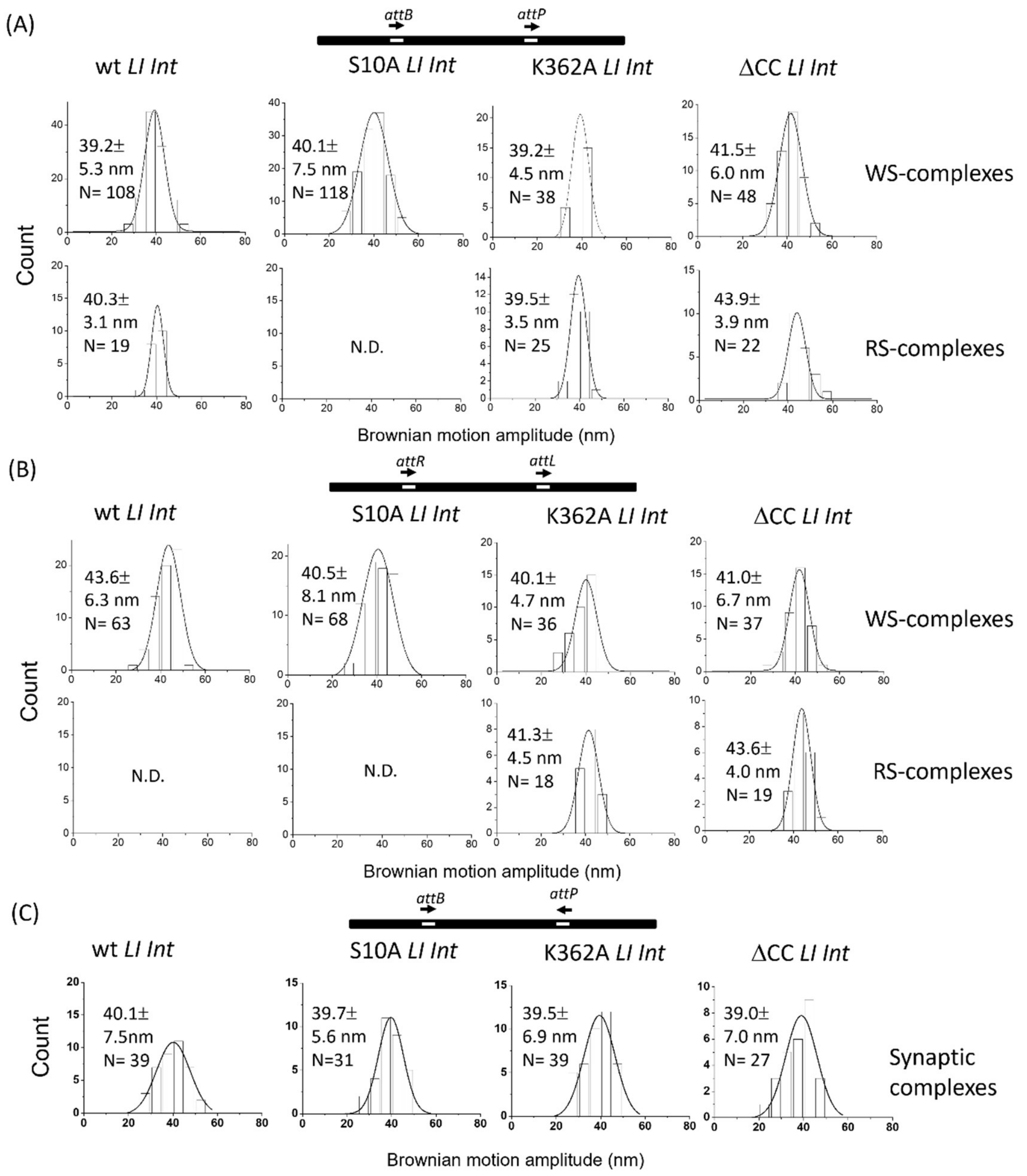


**Supplemental Figure 6.** *BM amplitude distributions of recombination-proficient (RS) and recombination-blocked (WS) synapses formed by* **A.** *attB* x *attP,* **B.***attL* x *attR in head-to-tail or* **C.** *attB* x *attP head-to-head orientations shown in above.* The BM amplitude distribution for the RS-complexes (recombination-proficient) and WS-complexes (recombination-blocked)) with wt *LI* integrases, S10A *LI* integrases, K362A *LI* integrases and ΔCC *LI* integrases. All fittings were performed in Origin 8.0, and all fitting values are listed in Table II.

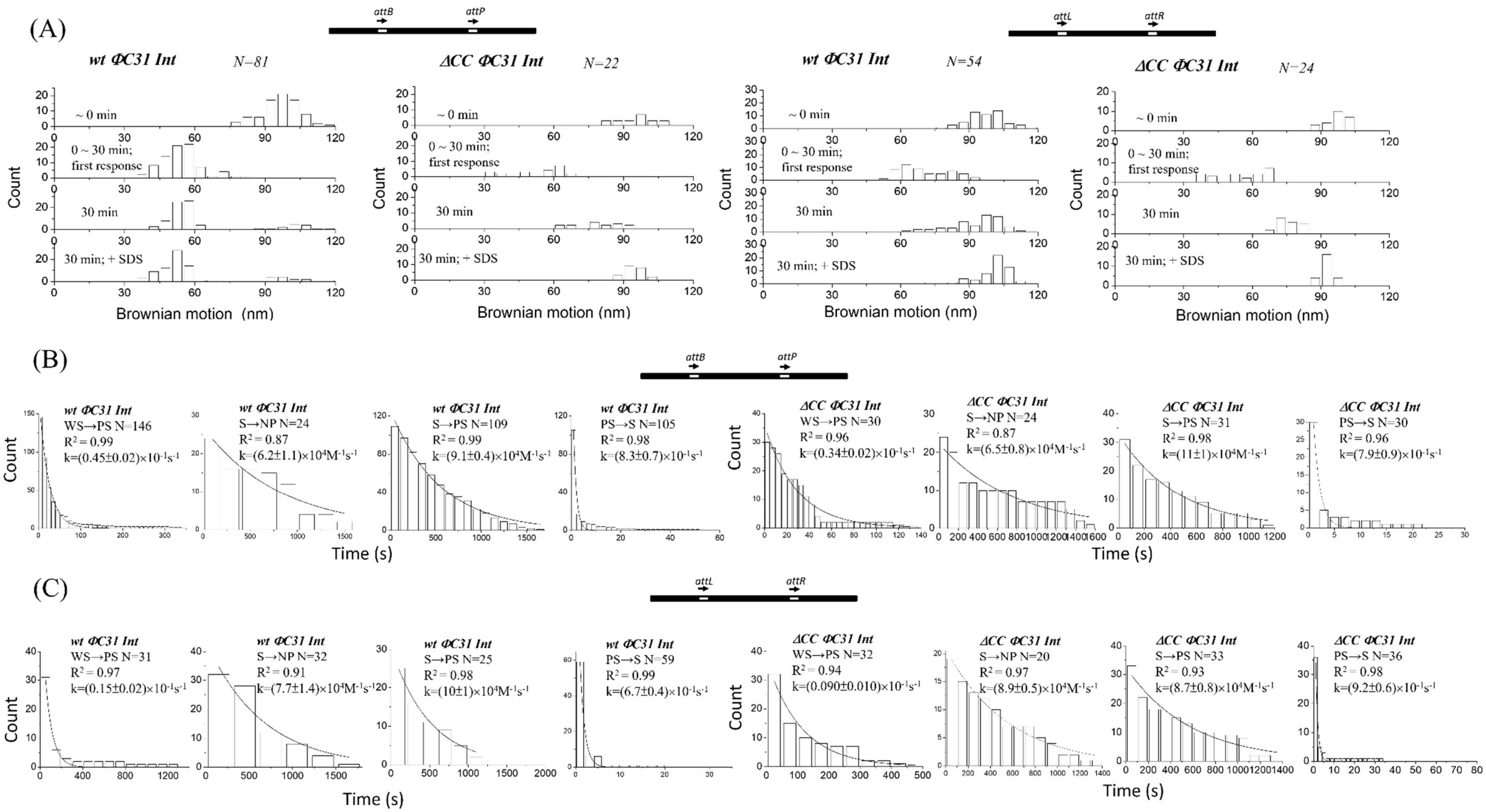
(A)
attB attP
attL attR
wt ΦC31 Int N=81
ΔCC ΦC31 Int N=22
wt ΦC31 Int N=54
ΔCC ΦC31 Int N=24
~ 0 min
0 ~ 30 min; first response
30 min
30 min; + SDS
Count
Brownian motion (nm)
(B)
wt ΦC31 Int WS→PS N=146 R² = 0.99 k=(0.45±0.02)×10⁻¹s⁻¹
wt ΦC31 Int S→NP N=24 R² = 0.87 k=(6.2±1.1)×10⁴M⁻¹s⁻¹
wt ΦC31 Int S→PS N=109 R² = 0.99 k=(9.1±0.4)×10⁴M⁻¹s⁻¹
wt ΦC31 Int PS→S N=105 R² = 0.98 k=(8.3±0.7)×10⁻¹s⁻¹
ΔCC ΦC31 Int WS→PS N=30 R² = 0.96 k=(0.34±0.02)×10⁻¹s⁻¹
ΔCC ΦC31 Int S→NP N=24 R² = 0.87 k=(6.5±0.8)×10⁴M⁻¹s⁻¹
ΔCC ΦC31 Int S→PS N=31 R² = 0.98 k=(11±1)×10⁴M⁻¹s⁻¹
ΔCC ΦC31 Int PS→S N=30 R² = 0.96 k=(7.9±0.9)×10⁻¹s⁻¹
Count
Time (s)
(C)
wt ΦC31 Int WS→PS N=31 R² = 0.97 k=(0.15±0.02)×10⁻¹s⁻¹
wt ΦC31 Int S→NP N=32 R² = 0.91 k=(7.7±1.4)×10⁴M⁻¹s⁻¹
wt ΦC31 Int S→PS N=25 R² = 0.98 k=(10±1)×10⁴M⁻¹s⁻¹
wt ΦC31 Int PS→S N=59 R² = 0.99 k=(6.7±0.4)×10⁻¹s⁻¹
ΔCC ΦC31 Int WS→PS N=32 R² = 0.94 k=(0.090±0.010)×10⁻¹s⁻¹
ΔCC ΦC31 Int S→NP N=20 R² = 0.97 k=(8.9±0.5)×10⁴M⁻¹s⁻¹
ΔCC ΦC31 Int S→PS N=33 R² = 0.93 k=(8.7±0.8)×10⁴M⁻¹s⁻¹
ΔCC ΦC31 Int PS→S N=36 R² = 0.98 k=(9.2±0.6)×10⁻¹s⁻¹
Count
Time (s)

**Supplemental Figure 7.** *The reaction behaviors of 1303 bp DNA molecule containing attB-attP or attL-attR sites in head-to-tail orientation in response to wt ϕC31 integrases and ΔCC ϕC31 integrases.* The BM amplitude distribution of DNA molecules (**A**) with *att*B-*att*P sites in head-to-tail orientation and *att*L-*att*R sites in head-to-tail orientation in response to the addition of *wt ϕC31* integrases *or ΔCC ϕC31* integrases. From top to bottom: just prior to the addition of integrase (-0 min), first response, at 30 min of incubation (just prior to SDS addition) and after SDS challenge, respectively. Kinetic analysis of 1303 bp DNA molecule containing (**B**) *att*B-*att*P sites or (C) *att*L-*att*R sites in response to *wt ϕC31 integrases or ΔCC ϕC31* integrases. All fittings were performed in Origin 8.0, and all fitting values are listed in Table I. The error is in 95.5% CL.

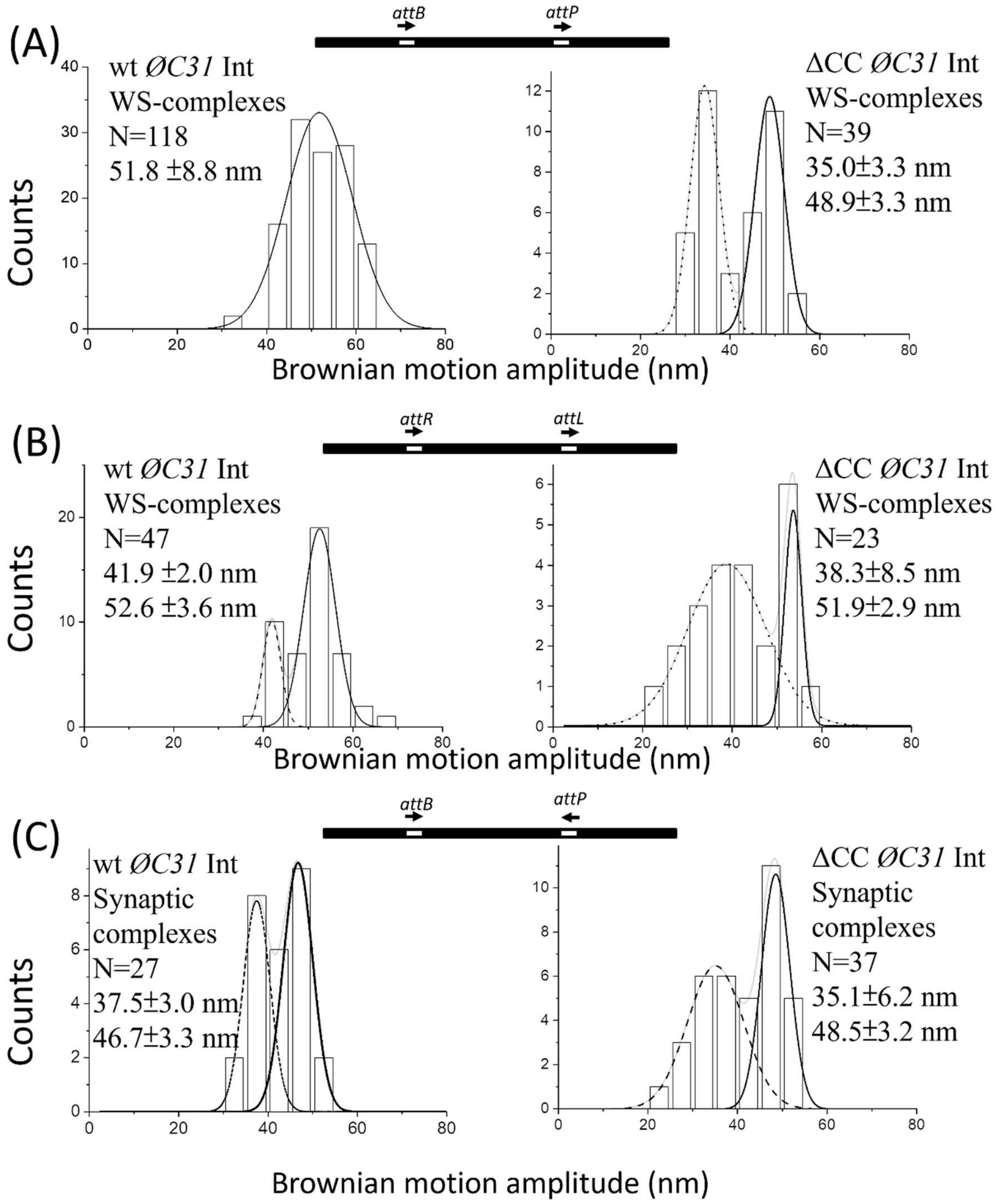


**Supplemental Figure 8.** *BM amplitude distributions of recombination-proficient (RS) and recombination-blocked (WS) synapses.* Synapses formed by **A.** *attB* x *attP*, **B**. *attL* x *attR* in head-to-tail or **C.** *attB* x *attP* head-to-head orientations shown above. The BM amplitude distribution for the RS-complexes (recombination-proficient) and WS-complexes (recombination-blocked)) with wt *ϕC31* integrases and ΔCC *ϕC31* integrases. All fittings were performed in Origin 8.0, and all fitting values are listed in Table II. The black dashed and black

solid lines represent the individual peak distributions, while the grey line represents the sum of the two peak distributions.

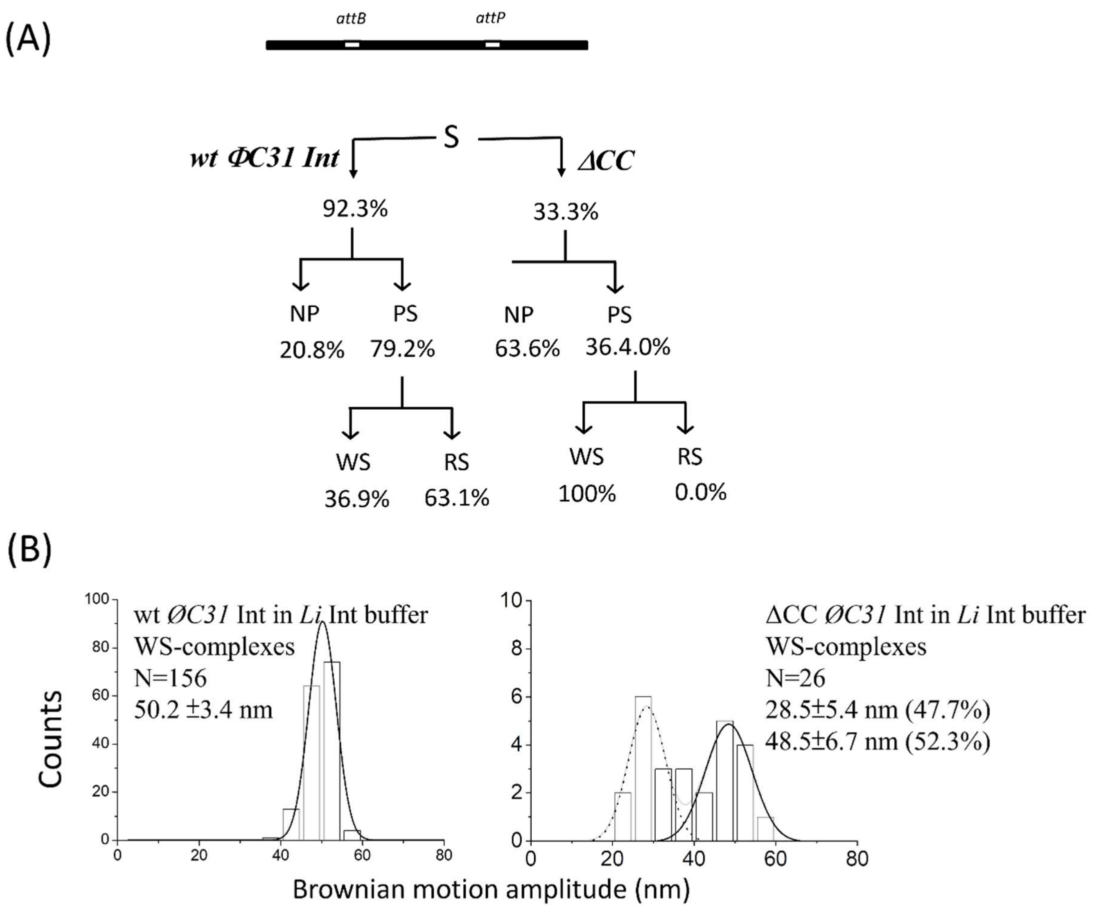


**Supplemental Figure 9.** *Analysis of 1303 bp DNA molecule containing attB-attP sites* in head-to-tail orientation *in response to ϕC31 integrases in the LI int buffer.* **A**. The reaction behaviors of *wt ϕC31* integrases and ΔCC *ϕC31* integrases. **B.** BM amplitude distributions of recombination-blocked (WS) synapses formed by *attB* x *attP* in head-to-tail orientations shown above. All fittings were performed in Origin 8.0. The black dashed and black solid lines represent the individual peak distributions, while the grey line represents the sum of the two peak distributions.

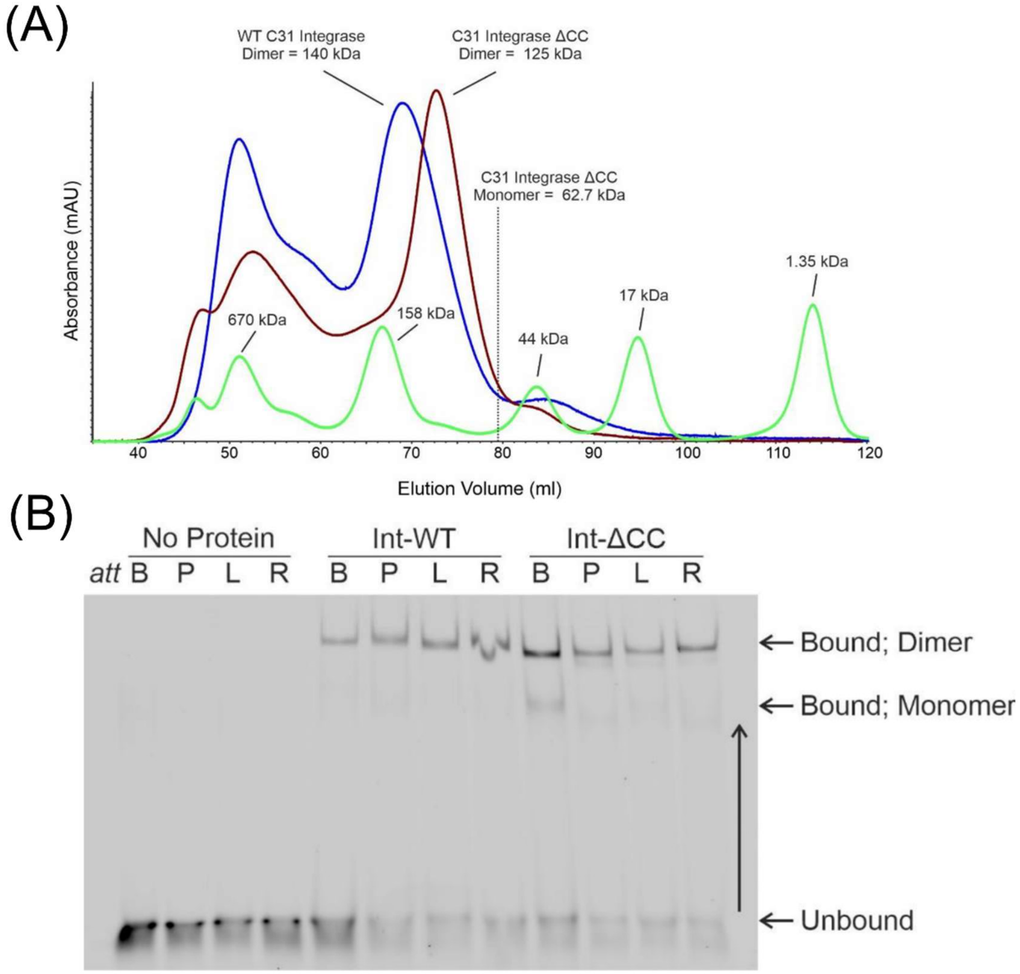
(A)
WT C31 Integrase
Dimer = 140 kDa
C31 Integrase ΔCC
Dimer = 125 kDa
C31 Integrase ΔCC
Monomer = 62.7 kDa
670 kDa
158 kDa
44 kDa
17 kDa
1.35 kDa
Absorbance (mAU)
Elution Volume (ml)
40
50
60
70
80
90
100
110
120
(B)
No Protein
Int-WT
Int-ΔCC
att
B
P
L
R
B
P
L
R
B
P
L
R
Bound; Dimer
Bound; Monomer
Unbound

**Supplemental Figure 10.** *Evaluation of the oligomeric state of ϕC31 wt and ΔCC integrase proteins in solution.* **A**. Analytical gel filtration of integrase proteins. Representative absorbance (280 nm) traces of Bio-Rad gel filtration molecular weight standard (green, Cat#1511901), wt ϕC31 integrase (blue) and ΔCC ϕC31 integrase (red) versus elution time from the column. Both integrase proteins appear to be dimers in solution. Absorbance values are omitted on the Y-axis because the traces were scaled differently to improve comparability. Approximate sizes of the standards are annotated on the graph. The relevant integrase peaks are labelled along with the theoretical sizes of respective dimers. The high MW peaks are likely to be aggregates due to the concentration process prior to loading. The dotted vertical line shows where the ΔCC ϕC31 Int monomer would be expected to elute. **B**. Electrophoretic mobility shift assay (EMSA) of ϕC31 integrase proteins with cognate *att* site DNA. AlexaFluor647-labelled oligonucleotides were annealed to an unlabelled complementary oligonucleotide to produce each 50 bp dsDNA substrates (*attB, attP, attL, attR*). The "No Protein" control lanes show a single high mobility DNA only band for all *att* site DNAs, whereas addition of wt ϕC31 integrases or ΔCC ϕC31 integrases leads to decreased DNA mobility for all DNA substrates. The major shifted DNA bands (equivalent to Complex II in McEwan *et al*. 2009) for both proteins are of almost equivalent mobility (the ΔCC ϕC31 integrases bands are slightly lower due to the smaller size of the protein). Both proteins also produce a minor band of intermediate mobility, presumably representing monomer bound to DNA (Complex I in McEwan *et al*. 2009), and this band is slightly more prominent for ΔCC ϕC31 integrase lanes.

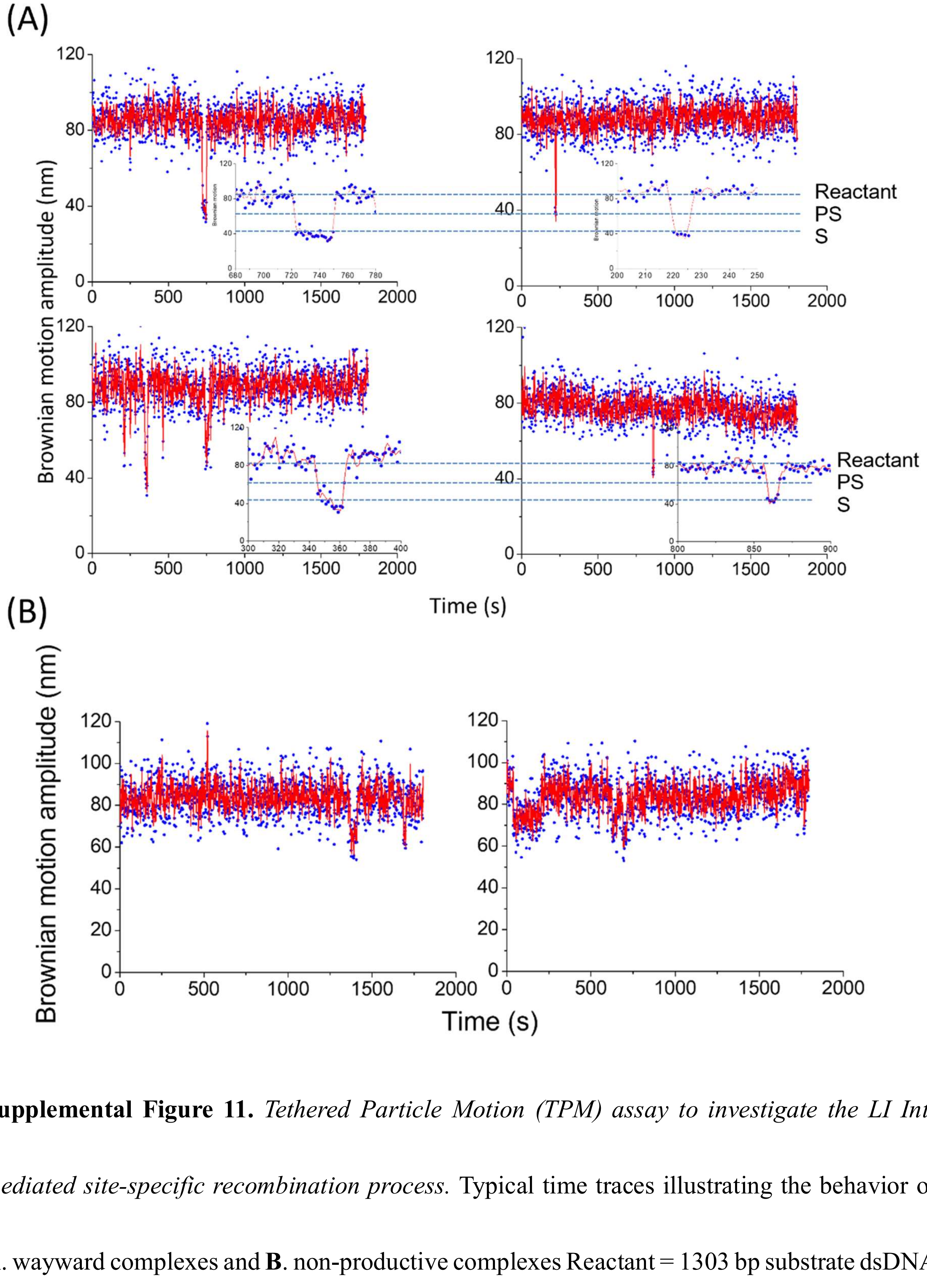


**Supplemental Figure 11.** *Tethered Particle Motion (TPM) assay to investigate the LI Int-mediated site-specific recombination process.* Typical time traces illustrating the behavior of **A**. wayward complexes and **B**. non-productive complexes Reactant = 1303 bp substrate dsDNA, PS = presynaptic complex, S = synaptic complex.

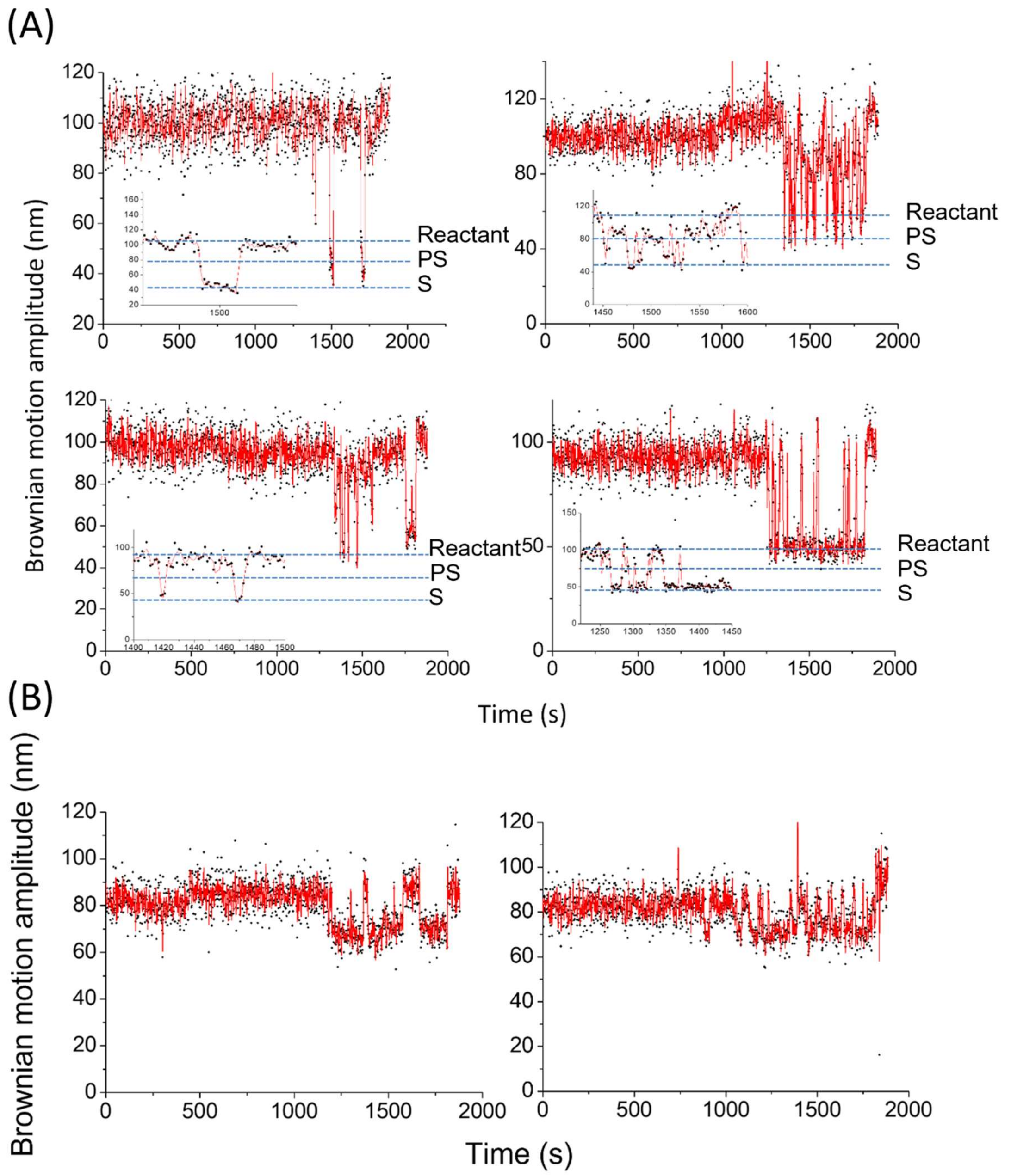


**Supplemental Figure 12.** *Tethered Particle Motion (TPM) assay to investigate $\phi$C31 Int-mediated site-specific recombination process.* Typical time traces illustrating the behavior of **A**. wayward complexes and **B**. non-productive complexes Reactant = 1303 bp substrate dsDNA, PS = presynaptic complex, S = synaptic complex.

**Supplementary Table I.** Substrate *att* sequences used for *ϕC31* int and *LI* int recombination assays

| | *att* site | DNA sequence |
|---|---|---|
| *ϕC31* | *attP* | 5’-GGAGTAGTGCCCCAACTGGGGTAACCTTTGAGTTCTCTCAGTTGGGGGCGTAGGGTC-3’<br>3’-CCTCATCACGGGGTTGACCCCATTGGAAACTCAAGAGAGTCAACCCCCGCATCCCAG-5’ |
| | *attB* | 5’-CCGCGGTGCGGGTGCCAGGGCGTGCCCTTGGGCTCCCCGGGCGCGTACTCCACCTCA-3’<br>3’-GGCGCCACGCCCACGGTCCCGCACGGGAACCCGAGGGGCCCGCGCATGAGGTGGAGT-5’ |
| | *attL* | 5-’CCGCGGTGCGGGTGCCAGGGCGTGCCCTTGAGTTCTCTCAGTTGGGGGCGTAGGGTC-3’<br>3’-GGCGCCACGCCCACGGTCCCGCACGGGAACTCAAGAGAGTCAACCCCCGCATCCCAG-5’ |
| | *attR* | 5’-GGAGTAGTGCCCCAACTGGGGTAACCTTTGGGCTCCCCGGGCGCGTACTCCACCTCA-3’<br>3’-CCTCATCACGGGGTTGACCCCATTGGAAACCCGAGGGGCCCGCGCATGAGGTGGAGT-5’ |
| *LI* | *attP* | 5’- TTGTTTAGTATCTCGTTATCTCTCGTTGGAGGGAGAAGAAACGGGATACCAAAAAT -3’<br>3’- AACAAATCATAGAGCAATAGAGAGCAACCTCCCTCTTCTTTGCCCTATGGTTTTTA -5’ |
| | *attB* | 5’- TGTAACTTTTTCGGATCGAGTTATGATGGACGTAAAGAGGGAACAAAGCATCTAAT -3’<br>3’- ACATTGAAAAAGCCTAGCTCAATACTACCTGCATTTCTCCCTTGTTTCGTAGATTA -5’ |
| | *attL* | 5’- TGTAACTTTTTCGGATCGAGTTATGATGGAGGGAGAAGAAACGGGATACCAAAAAT -3’<br>3’- ACATTGAAAAAGCCTAGCTCAATACTACCTCCCTCTTCTTTGCCCTATGGTTTTTA -5’ |
| | *attR* | 5’- TTGTTTAGTATCTCGTTATCTCTCGTTGGACGTAAAGAGGGAACAAAGCATCTAAT -3’<br>3’- AACAAATCATAGAGCAATAGAGAGCAACCTCCCTCTTCTTTGCCCTATGGTTTTTA -5’ |

**NB** Underlined bases are the central crossover region of the respective *att* sites.

**Supplementary Table II** Primer sequences used to obtain the experimental DNA sequences

| DNA substrate | Template | Primer sequence |
|---|---|---|
| *att*B/*att*P sites *in head-to-tail orientation* 1302 dsDNA | PL451*(parallel-BP)* | 5′-DigN- CCCGCCGCGCTTAATGCGC<br>5′-Bio- CCCGACTGGAAAGCGGGC |
| *att*B/*att*P sites *in head-to-head orientation* 1302 dsDNA | PL451 *(inverse-BP)* | 5′-DigN- CCCGCCGCGCTTAATGCGC<br>5′-Bio- CCCGACTGGAAAGCGGGC |
| *att*R/*att*L sites *in head-to-tail orientation* 1302 dsDNA | PL451*(parallel-RL)* | 5′-DigN- CCCGCCGCGCTTAATGCGC<br>5′-Bio- CCCGACTGGAAAGCGGGC |
| Single *att*P site 1302 ds DNA | PL451*(parallel-BP)* | 5′-DigN- CCGAAGGTAACTGGCTTC<br>5′-Bio- AGGGACTGGCTGCTATTGG |
| Single *att*R site 1302 ds DNA | PL451*(parallel-RL)* | 5′-DigN- CCGAAGGTAACTGGCTTC<br>5′-Bio- AGGGACTGGCTGCTATTGG |
| Single *att*B site 1302 ds DNA | PL451*(singel-attB)* | 5′-DigN- CCCGCCGCGCTTAATGCGC<br>5′-Bio- GGACTGGCTGCTATTGG |
| Single *att*L site 1302 ds DNA | PL451*(singel-attL)* | 5′-DigN- CCCGCCGCGCTTAATGCGC<br>5′-Bio- GGACTGGCTGCTATTGG |
| 1302 dsDNA | pBR322 | 5′-DigN- TGTGCCCAGTCATAGCC<br>5′-Bio- AGATGCGCCGCGTGCGGC |
| 551 dsDNA | PL451*(parallel-RL)* | 5′-DigN –CCCGCCGCGCTTAATGCGC<br>5′-Bio- TGTGCCCAGTCATAGCC |

**Supplementary Table III** Kinetics of recombination between *att* sites mediated by *wt* *ϕC31* integrase or ΔCC *ϕC31* integrase in the*ϕC31* Int and *LI* int buffer. The rate constants were determined by fitting the dwell times to a single exponential model.

| Reaction conditions | | | *buffer* | *k-NP$_f$* *($10^4 M^{-1}s^{-1}$)* | *k-PS$_f$* *($10^4 M^{-1}s^{-1}$)* | *k-RS$_f$* *($10^{-1}s^{-1}$)* | *k-WS$_f$* *($10^{-1}s^{-1}$)* | *k-WS$_d$* *($10^{-1}s^{-1}$)* | *k-NP$_d$* *($10^{-2}s^{-1}$)* | *k-PS$_d$* *($10^{-1}s^{-1}$)* |
|---|---|---|---|---|---|---|---|---|---|---|
| *attB-attP* | *ϕC31 integrase* | *wt* | *ϕC31 int* | 5.78±1.06 | 9.11±0.35 | 2.20 ±0.34 | 3.20±0.23 | 0.45±0.02 | 2.20±0.15 | 8.31±0.67 |
| | | | *LI int* | *(8.89±0.90)* | *(9.45±0.93)* | *(0.99±0.11)* | *(1.03±0.10)* | *(0.69±0.08)* | *(3.21±0.24)* | *(3.03±0.32)* |
| | | *ΔCC* | *ϕC31 int* | 6.21±0.57 | 8.88±0.55 | N.D. | 1.85±0.70 | 0.34±0.01 | 1.00±0.08 | 7.93±0.94 |
| | | | *LI int* | *(8.64±0.92)* | *(7.95±1.48)* | N.D. | *(0.67±0.04)* | *(0.72±0.05)* | *(2.89±0.18)* | *(4.41±1.53)* |
